# AGILE: a seamless phase I/IIa platform for the rapid evaluation of candidates for COVID-19 treatment: an update to the structured summary of a study protocol for a randomised platform trial letter

**DOI:** 10.1186/s13063-021-05458-4

**Published:** 2021-07-26

**Authors:** Gareth O. Griffiths, Richard FitzGerald, Thomas Jaki, Andrea Corkhill, Helen Reynolds, Sean Ewings, Susannah Condie, Emma Tilt, Lucy Johnson, Mike Radford, Catherine Simpson, Geoffrey Saunders, Sara Yeats, Pavel Mozgunov, Olana Tansley-Hancock, Karen Martin, Nichola Downs, Izabela Eberhart, Jonathan W. B. Martin, Cristiana Goncalves, Anna Song, Tom Fletcher, Kelly Byrne, David G. Lalloo, Andrew Owen, Michael Jacobs, Lauren Walker, Rebecca Lyon, Christie Woods, Jennifer Gibney, Justin Chiong, Nomathemba Chandiwana, Shevin Jacob, Mohammed Lamorde, Catherine Orrell, Munir Pirmohamed, Saye Khoo, on behalf of the AGILE investigators

**Affiliations:** 1 Southampton Clinical Trials Unit, University of Southampton, Southampton, Hampshire, UK; 2 NIHR Royal Liverpool and Broadgreen CRF, Liverpool University Hospitals NHS Foundation Trust, Liverpool, UK; 3 Lancaster University, Lancaster UK and MRC Biostatistics Unit, University of Cambridge, Cambridge, UK; 4 University of Liverpool, Liverpool, UK; 5 Liverpool School of Tropical Medicine, Liverpool, UK; 6 Royal Free London NHS Foundation Trust, London, UK; 7 University of the Witwatersrand, Johannesburg, South Africa; 8 Infectious Diseases Institute, Makerere University, Kampala, Uganda; 9 Desmond Tutu Health Foundation, University of Cape Town, Cape Town, South Africa

**Keywords:** COVID-19, SARS-CoV-2, Randomised controlled trial, Platform study, Master protocol, Phase I/II, Bayesian

## Abstract

**Background:**

There is an urgent unmet clinical need for the identification of novel therapeutics for the treatment of COVID-19. A number of COVID-19 late phase trial platforms have been developed to investigate (often repurposed) drugs both in the UK and globally (e.g. RECOVERY led by the University of Oxford and SOLIDARITY led by WHO). There is a pressing need to investigate novel candidates within early phase trial platforms, from which promising candidates can feed into established later phase platforms. AGILE grew from a UK-wide collaboration to undertake early stage clinical evaluation of candidates for SARS-CoV-2 infection to accelerate national and global healthcare interventions.

**Methods/design:**

AGILE is a seamless phase I/IIa platform study to establish the optimum dose, determine the activity and safety of each candidate and recommend whether it should be evaluated further. Each candidate is evaluated in its own trial, either as an open label single arm healthy volunteer study or in patients, randomising between candidate and control usually in a 2:1 allocation in favour of the candidate. Each dose is assessed sequentially for safety usually in cohorts of 6 patients. Once a phase II dose has been identified, efficacy is assessed by seamlessly expanding into a larger cohort. AGILE is completely flexible in that the core design in the master protocol can be adapted for each candidate based on prior knowledge of the candidate (i.e. population, primary endpoint and sample size can be amended). This information is detailed in each candidate specific trial protocol of the master protocol.

**Discussion:**

Few approved treatments for COVID-19 are available such as dexamethasone, remdesivir and tocilizumab in hospitalised patients. The AGILE platform aims to rapidly identify new efficacious and safe treatments to help end the current global COVID-19 pandemic. We currently have three candidate specific trials within this platform study that are open to recruitment.

**Trial registration:**

EudraCT Number: 2020-001860-27 14 March 2020

ClinicalTrials.gov Identifier: NCT04746183 19 February 2021

ISRCTN reference: 27106947

**Supplementary Information:**

The online version contains supplementary material available at 10.1186/s13063-021-05458-4.

## Background

In 2019, a novel coronavirus disease (COVID-19) emerged in Wuhan, China, which is caused by severe acute respiratory syndrome coronavirus 2 (SARS-CoV-2) [1]. Many patients do not progress to severe disease, but as COVID-19 spread across the world, vast numbers of patients have been hospitalised with respiratory failure, a significant proportion of whom have become critically unwell and died [2–4]. The progression from initial symptoms, most commonly fever, fatigue and cough, to respiratory failure requiring oxygen support or mechanical ventilation occurs several days after the onset of symptoms [2]. While treatment options such as dexamethasone, remdesivir and tocilizumab are now available for certain hospitalised patients with severe disease, these are repurposed medicines [5, 6]. AGILE is unique as it provides a platform for assessing novel therapeutic agents. There is also a distinct lack of available treatment options for the early phase of infection which may help prevent progression to severe disease [7].

Given the rapid and global spread of COVID-19 and the emergence of new variants in the UK, South Africa, India and Brazil, a robust but rapid assessment of potential treatments is needed [8]. The AGILE platform aims to identify new candidates with promising evidence of efficacy that can be further evaluated in subsequent larger phase IIb/III trials. Having one master protocol ensures different candidates are evaluated in the same consistent manner and enables more efficient introduction of new trials for new candidates.

## Methods/design

### Objectives

Phase I—To determine the optimal dose of each candidate (or combination of candidates) entered into the platform. Phase II—To determine the efficacy and safety of each candidate entered into the platform, compared to the current Standard of Care (SoC), and recommend whether it should be evaluated further in later phase II and III platforms.

### Trial design

AGILE is a multicentre, multi-candidate, multi-dose, multi-stage, randomised phase I/IIa Bayesian adaptive platform trial to determine the safety and efficacy of multiple candidate agents for the treatment of COVID-19. The multi-candidate design allows many candidates to be tested simultaneously (while potentially sharing control group data, provided they are at least contemporaneously recruited), in order to increase efficiency compared to multiple single-candidate studies. The multi-dose feature allows progression beyond the licensed dose dependent on adequate safety and tolerability data and promising efficacy. The multi-stage feature allows for pre-specified analyses that can be used to determine dropping of ineffective doses or candidates or recommending doses or candidates for further phase II/III testing, further increasing efficiency of the study. The adaptive platform design allows for the removal of unpromising candidates, promising candidates to be recommended for further testing in external phase II/III studies, and the addition of new potential treatments to be added during the trial. Candidates will be added into the AGILE platform study via candidate specific trial (CST) protocols to the master protocol.

### Participants

Patient populations can vary between CSTs, but the main eligibility criteria include adult patients (≥ 18 years) who have laboratory-confirmed infection with SARS-CoV-2. We will include both severe and mild-moderate patients as well as healthy volunteers defined as follows: group A (severe disease)—patients with WHO Clinical Progression Scale [9] of grades 5 (hospitalised, oxygen by mask or nasal prongs), 6 (hospitalised, non-invasive ventilation or high flow oxygen), 7 [hospitalised, intubation and mechanical ventilation, arterial oxygen partial pressure (PaO_2_)/fractional inspired oxygen (FiO_2_) ≥ 150 or peripheral capillary oxygen saturation (SpO_2_)/FiO_2_ ≥ 200], 8 [hospitalised mechanical ventilation PaO_2_/FiO_2_ < 150 (SpO_2_/FiO_2_ < 200) or vasopressors] or 9 (hospitalised, mechanical ventilation pO_2_/FiO_2_ < 150 and vasopressors, dialysis or ECMO). Group B (mild-moderate disease)—ambulant or hospitalised patients with SpO_2_ > 94% room air. If any CSTs include participants from the community setting, the CST protocol will clarify whether patients with suspected SARS-CoV-2 infection (in addition to laboratory-confirmed infection) are also eligible. Group C (healthy volunteers)—to be defined in each CST protocol. Participants are currently being recruited from the UK, but we intend to open up recruitment at international sites later in 2021.

### Intervention and comparator

The comparator is the current SoC, and in some CSTs, placebo is added to the current SoC. Candidates that prevent uncontrolled cytokine release, prevention of viral replication, and other anti-viral treatment strategies are at various stages of development for inclusion into AGILE. Other CSTs will be added over time. There is not a set limit on the number of CSTs we can include within the AGILE master protocol, and we will upload each CST into this publication as each opens to recruitment.

### Main outcomes

Phase I—Dose limiting toxicities using Common Terminology Criteria for Adverse Events (CTCAE) Version 5 Grade ≥ 3 adverse events. Phase II—Agreed on a CST basis depending on mechanism of action of the candidate and patient population, but may include time to clinical improvement of at least 2 points on the WHO Clinical Progression Scale [measured up to 29 days from randomisation], progression of disease (SpO_2_ < 92%) or hospitalisation or death, or change in time-weighted viral load [measured up to 29 days from randomisation].

### Randomisation and blinding

Each candidate will be evaluated in its own CST, either as an open label single arm healthy volunteer study or in patients, randomising between candidate and control. Randomisation varies with each CST, but the default is 2:1 allocation in favour of the candidate to maximise early safety data. Each dose will be assessed sequentially for safety in cohorts of patients; the default size is 6 patients per cohort. Once a phase II dose has been identified, we will assess efficacy by seamlessly expanding into a larger cohort.

### Numbers to be randomised (sample size)

Sample size varies between CSTs. Simulations have shown that around 16 participants are necessary to determine futility or promise of a candidate at a given dose (in efficacy evaluation alone) and between 32 and 40 participants are required across the dose-finding and efficacy evaluation when capping the maximum number of participants contributing to the evaluation of a treatment at 40. Each CST protocol will give details on the sample size needed for that candidate and a justification for any CST needing more than 40 participants.

### Full protocol

The full AGILE master protocol is attached as an additional file, accessible from the Trials website www.agiletrial.net (Additional file 1). In the interest of expediting dissemination of this material, the familiar formatting has been eliminated; this addendum is an update to the original letter and serves as a summary of the key elements of the full master protocol and trial status of active CSTs.

## Discussion

AGILE is the first and only phase I/IIa national clinical trial platform in the UK to determine the safety and efficacy of promising candidate agents for COVID-19. It bridges the gap between preclinical development and later phase trials, so that potential new treatments can pass through the phases of drug development in a rapid and safe manner. An independent COVID-19 Therapeutics Advisory Panel (UK-CTAP) will evaluate and prioritise candidate agents for inclusion in the study. The design of AGILE allows for the assessment of multiple candidates at different doses, with the ability to add candidates as they are identified or drop them as their evaluation is completed. Promising candidates will move to an external trial for further evaluation in the phase II/III setting. AGILE is completely flexible and each CST design is catered to the requirement of the candidate whereby population, primary endpoint and sample size can be amended. AGILE has full regulatory approval [by the UK MHRA, ethics and HRA and the WHO Research Ethics Review Committee (ERC)] and has three CSTs currently open to recruitment with a number of others at various stages of development. AGILE has the potential to rewrite the treatment landscape for COVID-19 and shape our response to future global pandemics.

## Trial status

### AGILE master protocol

The AGILE master protocol Version 9 (17 February 2021) has full regulatory approval and utilises several digital technology solutions, including Medidata’s Rave Electronic Data Capture (EDC), Randomization and Trial Supply Management (RTSM) for randomisation and drug distribution, patient eConsent on iPads and electronic Clinical Outcome Assessment (eCOA) to collect patient reported outcome measures via an App on the participants smart phones.

### CST-2: a randomised, multicentre, seamless, adaptive, phase I/II platform study to determine the optimal dose, safety and efficacy of Molnupiravir (EIDD-2801) for the treatment of COVID-19

CST-2 has full regulatory approval, has completed phase I and has successfully determined a dose for phase II evaluation. Phase II recruitment is ongoing following a planned interim analysis by an Independent Data Monitoring Committee. Molnupiravir is being evaluated in adult ambulant patients with laboratory confirmed COVID-19, who are within 5 days of symptom onset in the UK. Molnupiravir (EIDD-2801) is the 5′-isopropyl ester prodrug of the broadly active, direct-acting antiviral ribonucleoside analogue EIDD-1931. Molnupiravir is being developed for the treatment of infections caused by RNA viruses, specifically for COVID-19 and other coronavirus infections, influenza and Venezuelan equine encephalitis virus [10, 11].

### CST-3A: a multicentre, adaptive, phase I trial to determine the optimal dose, safety and efficacy of nitazoxanide for the treatment of COVID-19

CST-3A has full regulatory approval and has recently completed recruitment. Nitazoxanide is being evaluated in healthy adults in the UK. Nitazoxanide is a thiazolide FDA-approved antiparasitic medicine used for the treatment of cryptosporidiosis and giardiasis diarrhoea but has been studied previously for viruses including influenza [12]. However, preclinical candidate evaluation by AGILE investigators using data from in vitro antiviral activity and historical human pharmacokinetic studies strongly suggests that doses higher than those approved will be required for durable antiviral activity in SARS-CoV-2 infection [13, 14]. N.B. Once CST-3A has completed in the UK, it is planned that a CST-3B phase Ib/II trial will open in South Africa and Uganda in adult ambulant patients with laboratory-confirmed COVID-19.

### CST-5: a randomised, multicentre, seamless, adaptive, phase I/II platform study to determine the phase II dose of VIR-7832, and evaluate the safety and efficacy of VIR-7831 and VIR-7832 for the treatment of COVID-19

CST-5 has full regulatory approval and phase I is currently recruiting. VIR-7832 and VIR-7831 are being evaluated in adult ambulant patients with laboratory-confirmed COVID-19, who are within 5 days of symptom onset in the UK. VIR-7832 and VIR-7831 are both human monoclonal antibodies that neutralise COVID-19 by targeting the receptor-binding domain of the SARS-CoV-2 Spike (S) glycoprotein [15]. The binding of this domain inhibits the entry of the SARS-CoV-2 virus into the host cell. VIR-7832 includes the “XX2” modification in the fragment crystallisable domain. The XX2 modification is designed to enhance effector functions, abrogate C1q binding and elicit enhanced T cell and antibody responses [16].

## Supplementary Information

**Additional file 1..** AGILE Master Protocol Version 9 17-Feb-2021

## Data Availability

When the database from each CST protocol is locked, analysed and published, we will make the data available to the academic community via a request to the AGILE Data Sharing Committee (agile@soton.ac.uk).

## Abbreviations

COVID-19: Coronavirus disease 2019
CST: Candidate specific trial
CTCAE v5: Common Terminology Criteria for Adverse Events Version 5
eCOA: Electronic Clinical Outcome Assessment
EDC: Electronic Data Capture
ERC: Ethics Review Committee
FiO_2_: Fractional inspired oxygen
PaO_2_: Arterial oxygen partial pressure
RTSM: Randomization and Trial Supply Management
SARS-CoV-2: Severe acute respiratory syndrome coronavirus 2
SoC: Standard of Care
SpO_2_: Peripheral capillary oxygen saturation
UK-CTAP: UK COVID-19 Therapeutics Advisory Panel
