# Supplementary material for: AGILE: a seamless phase I/IIa platform for the rapid evaluation of candidates for COVID-19 treatment: an update to the structured summary of a study protocol for a randomised platform trial letter
Source: Trials. 2021 Jul 26;22:487. doi: 10.1186/s13063-021-05458-4 (PMC8311065; doi:10.1186/s13063-021-05458-4)
Supplement: Supplementary file 1 — Additional file 1.. AGILE Master Protocol Version 9 17-Feb-2021 [file 13063_2021_5458_MOESM1_ESM.pdf]

AGILE MASTER PLATFORM PROTOCOL

Seamless Phase I/IIa Platform for the Rapid Evaluation of Candidates for COVID-19 treatment

Version FINAL VERSION 9 17 FEB 2021

SPONSOR: University of Liverpool
COORDINATING CENTRES:
UK Sites - Southampton Clinical Trials Unit (SCTU)
International Sites – Liverpool School of Tropical Medicine, Global Health Trials Unit (GHTU)

Eudract Number:	2020-001860-27
Clinicaltrials.gov number:	NCT04746183
ISRCTN reference:	27106947
Ethics reference number:	20/WM/0136
Sponsor reference number:	UoL001542
ICD-10	U07.1 COVID-19, U0.71 COVID-19

Protocol authorised by:

**Name:**	Saye Khoo	**Role:**	Chief Investigator
**Signature:**		**Date:**	
**Name:**	Gareth Griffiths	**Role:**	Director of SCTU
**Signature:**		**Date:**	
**Name:**	Neil French	**Role:**	On behalf of Sponsor
**Signature:**		**Date:**	

MAIN TRIAL CONTACT

Chief Investigator: Professor Saye Khoo, Professor of Molecular and Clinical Pharmacology

Email: S.H.Khoo@liverpool.ac.uk

For general trial and clinical queries e.g. participant queries, trial supplies, data collection, please contact in the first instance:

AGILE Trial Manager: agile@soton.ac.uk Tel: 023 8120 5154

Address:	Southampton Clinical Trials Unit<br>Southampton General Hospital<br>Tremona Road<br>SOUTHAMPTON<br>SO16 6YD	Tel:<br>Fax:<br>Email:<br>Web:	023 8120 5154<br>0844 774 0621<br>ctu@soton.ac.uk

TRIAL WEBSITE www.agiletrial.net

SPONSOR

University of Liverpool is the research sponsor for this trial. For further information regarding sponsorship conditions, please contact:

Address: Clinical Directorate
4th Floor Thompson Yates Building,
Faculty of Health and Life Sciences
University of Liverpool
Liverpool L69 3GB
Tel: 0151 794 8373 Email: sponsor@liverpool.ac.uk

CO-INVESTIGATOR(S)

Co-Investigators can be contacted via the Trial Coordination Centre.

FUNDER

Each CST will secure funding with additional financial support from the NIHR Southampton Clinical Trials Unit.

Protocol Information

This protocol describes the AGILE trial and provides information about procedures for entering participants. The protocol should not be used as a guide for the treatment of other non-trial participants; every care was taken in its drafting, but corrections or amendments may be necessary. These will be circulated to investigators in the trial, but in accordance with sponsor requirements, sites entering participants for the first time are advised to contact the appropriate coordinating centre (either Southampton Clinical Trials Unit or the Global Health Trials Unit) to confirm they have the most recent version.

Compliance

This trial will adhere to the principles of Good Clinical Practice (GCP). It will be conducted in compliance with the protocol, the current Data Protection Regulations and all other regulatory requirements, as appropriate.

THIS MASTER PROTOCOL DETAILS THE FRAMEWORK FOR THE AGILE PLATFORM TRIAL.

PLEASE REFER TO THE INDIVIDUAL CST PROTOCOL FOR AGILE INTERNATIONAL TRIALS (OUTSIDE UK) WHERE ADDITIONAL NATIONAL REGULATORY AND ETHICAL REQUIREMENTS WILL BE DETAILED AND TAKE PRIMACY.

ANY DIFFERENCES BETWEEN EACH CST AND THE MASTER WILL BE HIGHLIGHTED IN THE CST PROTOCOL. WHERE THERE ARE DIFFERENCES, THE CST PROTOCOL TAKES PRECEDENCE AND SHOULD BE FOLLOWED.

TABLE OF CONTENTS

LIST OF ABBREVIATIONS 6
KEYWORDS 6
1 PROTOCOL SUMMARY 7
1.1 TRIAL SYNOPSIS 7
1.2 TRIAL SCHEMA 11
1.3 EXAMPLE SCHEDULE OF OBSERVATIONS AND PROCEDURES 12
2 INTRODUCTION 14
2.1 BACKGROUND AND RATIONALE 14
2.2 RISK BENEFITS FOR CURRENT TRIAL 16
3 OBJECTIVES AND ENDPOINTS 17
4 STUDY DESIGN 18
4.1 OVERALL DESIGN 19
4.2 TRIAL PHASES 19
4.3 JUSTIFICATION FOR DOSE 21
4.4 DEFINITION OF END OF TRIAL 21
5 SELECTION AND ENROLMENT OF PARTICIPANTS 21
5.1 INFORMED CONSENT 21
5.2 INCLUSION CRITERIA 23
5.3 EXCLUSION CRITERIA 23
5.4 SCREEN FAILURES 24
5.5 CONTRACEPTION 24
5.6 REGISTRATION / RANDOMISATION PROCEDURES 25
6 STUDY TREATMENT 25
7 DISCONTINUATION OF STUDY TREATMENT AND PATIENT DISCONTINUATION/WITHDRAWAL 25
7.1 TRIAL DISCONTINUATION 25
7.2 WITHDRAWAL 25
7.3 LOST TO FOLLOW UP 26
8 STUDY ASSESSMENTS AND PROCEDURES 26
8.1 SCREENING PROCEDURES 26
8.2 TRIAL PROCEDURES 27
8.3 PHARMACOKINETICS AND PHARMACODYNAMICS 30
8.4 DEVIATIONS AND SERIOUS BREACHES 31
9 SAFETY 31
9.1 DEFINITIONS 31
9.2 REPORTING WINDOWS 32
9.3 ADVERSE EVENT TERM AND SEVERITY GRADE 32
9.4 SERIOUSNESS 32
9.5 CAUSALITY 33
9.6 EXPECTEDNESS - SEE CANDIDATE-SPECIFIC TRIAL PROTOCOL 33
9.7 REPORTING PROCEDURES 33
9.8 SCTU RESPONSIBILITIES FOR SAFETY REPORTING TO REC 34
9.9 SCTU RESPONSIBILITIES FOR SAFETY REPORTING TO MHRA 35
10 STATISTICS AND DATA ANALYSES 35
10.1 METHOD OF RANDOMISATION 35
10.2 SAMPLE SIZE 35
10.3 STATISTICAL ANALYSIS PLAN (SAP) 35
11 REGULATORY 37
11.1 CLINICAL TRIAL AUTHORISATION 37
12 ETHICAL CONSIDERATIONS 37
12.1 CONFIDENTIALITY 37
13 SPONSOR 37

13.1 INDEMNITY 37
13.2 FUNDING 38
14 TRIAL OVERSIGHT GROUPS 38
14.1 TRIAL MANAGEMENT GROUP (TMG) 38
14.2 TRIAL STEERING COMMITTEE (TSC) 38
14.3 DATA MONITORING AND ETHICS COMMITTEE (DMEC) 38
14.4 CANDIDATE-SPECIFIC TRIAL SAFETY REVIEW COMMITTEE (SRC) 38
15 DATA MANAGEMENT 39
16 DATA SHARING REQUESTS FOR RESULTS THAT ARE AVAILABLE IN THE PUBLIC DOMAIN 39
17 MONITORING 40
17.1 CENTRAL MONITORING 40
17.2 CLINICAL SITE MONITORING 40
17.3 SOURCE DATA 40
17.4 AUDITS AND INSPECTIONS 40
18 RECORD RETENTION AND ARCHIVING 40
19 PUBLICATION POLICY 41
20 REFERENCES 42
21 CANDIDATE-SPECIFIC TRIAL PROTOCOLS 42
22 SUMMARY OF SIGNIFICANT CHANGES TO THE PROTOCOL 42

LIST OF ABBREVIATIONS

AE	Adverse Event
AR	Adverse Reaction
COVID-19	Coronavirus-Induced Disease
CI	Chief Investigator
CRF	Case Report Form
CRM	Continual Reassessment Method
CST	Candidate-Specific Trial
CTA	Clinical Trial Authorisation
CTCAE	Common Terminology Criteria for Adverse Events
DMEC	Data Monitoring and Ethics Committee
DMP	Data Management Plan
ECMO	Extracorporeal Membrane Oxygenation
GCP	Good Clinical Practice
GHTU	Global Health Trials Unit
IB	Investigator Brochure
IMP	Investigational Medicinal Product
IMV	Invasive Mechanical Ventilation
ISF	Investigator Site File
MedDRA	Medical Dictionary for Regulatory Activities
MHRA	Medicines and Healthcare products Regulatory Agency
NCI	National Cancer Institute
NEWS	National Early Warning Score
PD	Pharmacodynamics
PK	Pharmacokinetics
RDEE	Recommended Dose for Efficacy Evaluation
REC	Research Ethics Committee
RTSM	Randomisation and Trial Supply Management (RTSM)
SAE	Serious Adverse Event
SAP	Statistical Analysis Plan
SAR	Serious Adverse Reaction
SARS	Severe Acute Respiratory Syndrome
SARS-CoV-2	Severe Acute Respiratory Syndrome Coronavirus 2
SmPC	Summary of Product Characteristics
SCTU	Southampton Clinical Trials Unit
SRC	Safety Review Committee
SUSAR	Suspected Unexpected Serious Adverse Reaction
TiTE	Time-to-Event
TMF	Trial Master File
TMG	Trial Management Group
TSC	Trial Steering Committee
UK-CTAP	COVID-19 Therapeutics Advisory Panel

KEYWORDS
COVID-19; SARS coronavirus 2; SARS-CoV-2; Phase I; Phase II; Platform trial

1 PROTOCOL SUMMARY

1.1 TRIAL SYNOPSIS

Short title:	AGILE
Full title:	Seamless Phase I/IIa Platform for the Rapid Evaluation of Candidates for COVID-19 treatment

Phase:	Seamless Phase I/II
Population:	Adult patients (≥18 years) who have laboratory-confirmed infection with severe acute respiratory syndrome coronavirus 2 (SARS-CoV-2).<br><br>We will include both severe and mild-moderate patients defined according to the WHO Clinical Progression Scale[8] as follows:<br><br>**Group A (severe disease)**<br>Patients with clinical status of Grades 5 (hospitalised, oxygen by mask or nasal prongs), 6 (hospitalised, non-invasive ventilation or high flow oxygen), 7 (hospitalised, intubation and mechanical ventilation, pO_2/FiO_2 ≥150 or SpO_2/FiO_2 ≥200), 8 (hospitalised mechanical ventilation pO_2/FiO_2 <150 (SpO_2/FiO_2 <200) or vasopressors or 9 (hospitalised, mechanical ventilation pO_2/FiO_2 <150 and vasopressors, dialysis or ECMO).<br><br>**Group B (mild-moderate disease)**<br>• Ambulant or hospitalised patients with peripheral capillary oxygen saturation (SpO_2) >94% RA<br>• N.B. If any Candidate Specific Trials (CST) are included in the community setting, the CST protocol will clarify whether patients with suspected SARS-CoV-2 infection are also eligible (e.g. ICD-10 U0.71 COVID-19).<br><br>We will also include healthy volunteers as a separate group:<br><br>**Group C (Healthy Volunteers)**
Primary Objective:	**Phase I** - To determine the optimal dose of each candidate (or combination of candidates) where applicable<br>**Phase II** - To determine the efficacy and safety of each candidate and recommend whether it should be evaluated further
Trial Design:	AGILE is an international multicentre, multi-arm, multi-dose, multi-stage, adaptive, seamless phase I/II Bayesian randomised platform trial to determine the optimal dose, activity and safety of multiple candidate agents for the treatment of COVID-19.<br><br>This study allows for the assessment of many candidates at different doses, with the ability to add candidates as they are identified or drop them as their evaluation is completed. Promising candidates will move to an external trial for further evaluation in the phase II/III setting.

	Each candidate will be evaluated in its own trial, either as an open label single arm healthy volunteer study or in patients, randomising between candidate and control with 2:1 allocation in favour of the candidate. Each dose will be assessed for safety sequentially in cohorts of 6 patients. Once a phase II dose has been identified we will assess efficacy by seamlessly expanding into a larger cohort.<br><br>**Sample size:** Simulations have shown that an average of n=16-18 participants are required for the phase I stage of each candidate trial, with an average of 16 patients additionally required for the phase II stage.<br><br>Each candidate trial will continue to recruit until the posterior probability that the hazard ratio of time to clinical improvement is >1 is less than 33% (i.e. evidence of a futility for that candidate) or if the probability of the hazard ratio being larger than 1 is greater than 80% (i.e. evidence of efficacy for that candidate).<br><br>AGILE is completely flexible in that the core design in the master protocol (as has been explained above) can be adapted for each candidate based on prior knowledge of the candidate – i.e. population, primary endpoint and sample size can be amended. This will be detailed in each candidate-specific trial protocol of the master protocol.
**Investigational Medicinal Product/s:**	An independent COVID-19 Therapeutics Advisory Panel (UK-CTAP)will evaluate and prioritise candidate agents for inclusion in the study. Candidate-specific trial (CST) protocols will outline full details of each candidate trial.

**Primary Trial Endpoints:**	**Co-primary endpoints:**<br><br>**For dose finding (phase I)**<br>• Dose limiting toxicities (Safety and Tolerability of drug under study – CTCAE v5 Grade ≥3 adverse events)<br><br>**For efficacy evaluation (phase II)** one of the following depending on the population the candidate is being evaluated in:<br><br>• **Group A (severe disease)**<br>Time to clinical improvement: Improvement will be determined according to the WHO Clinical Progression Scale[8]. Improvement, defined by a minimum 2-step change in the scale, will be analysed as time to event, measured up to 29 days from randomisation.<br><br>• **Group B (mild-moderate disease)**<br>Pharmacodynamics of drug defined as time to negative viral titres in nose and/or throat swab, measured up to 15 days from randomisation.<br><br>• **Group C (healthy volunteers)**<br>Pharmacokinetics, safety and tolerability of candidate

**Secondary Trial Endpoints:**	To include:<br>• Safety: adverse events and serious adverse events<br>• Change in viral titre (nose and/or throat) over time<br>• Overall mortality<br>• Time to discharge<br>• Admission/readmission to hospital (for ambulant participants at home<br>• Proportion of patient discharged<br>• Treatment adherence<br>• NEWS2/qSOFA<br>• Clinical improvement over time<br>• Admission to ICU<br>• Duration of oxygen use and oxygen free days<br>• Duration of mechanical ventilation and mechanical ventilation free days<br>• The ratio of the oxygen saturation to fractional inspired oxygen concentration (SpO_2/FiO_2)<br>• Qualitative and quantitative PCR for SARS-CoV-2 in nose and/or throat swab<br>• Biomarkers for response
**Total Number of Sites:**	UK - Up to 23 NIHR Clinical Research Facilities/NHS sites.<br>International: Up to 10 sites<br>Site selection will include (but not be limited to) consideration of the following criteria for each CST:<br>• Established research teams undertaking trials to GCP compliance<br>• Capability and experience in early-phase clinical trials<br>• Previous experience in first-in-human trial conduct (as applicable)<br>• Capability of the unit to undertake overnight stays (as applicable)<br>• Capability of the unit to be divided between Covid and non-Covid work with capability to divide the unit staffing into appropriate teams to safely deliver care<br>• Capability to risk assess staff to ensure safe to work with Covid patients<br>• Potential to develop community recruitment strategy with established links to general practice network<br>• Capability and experience in rich PK sampling<br>• Geographical considerations determined by local Covid outbreaks<br>• Site teams with experience in securing import licenses for IMP (as applicable) *<br>• Sites already known to the Sponsor*<br>• Sites already known to funder.<br><br>**Required for due diligence as site visits and audits cannot be undertaken in the current pandemic.*<br><br>Before the study can be initiated, the prerequisites for conducting the study must be confirmed and the organisational preparations

	made with the trial centre. The suitability of the Investigator's research team, technical facilities and availability of eligible participants at the trial centre must be ensured.

1.2 TRIAL SCHEMA

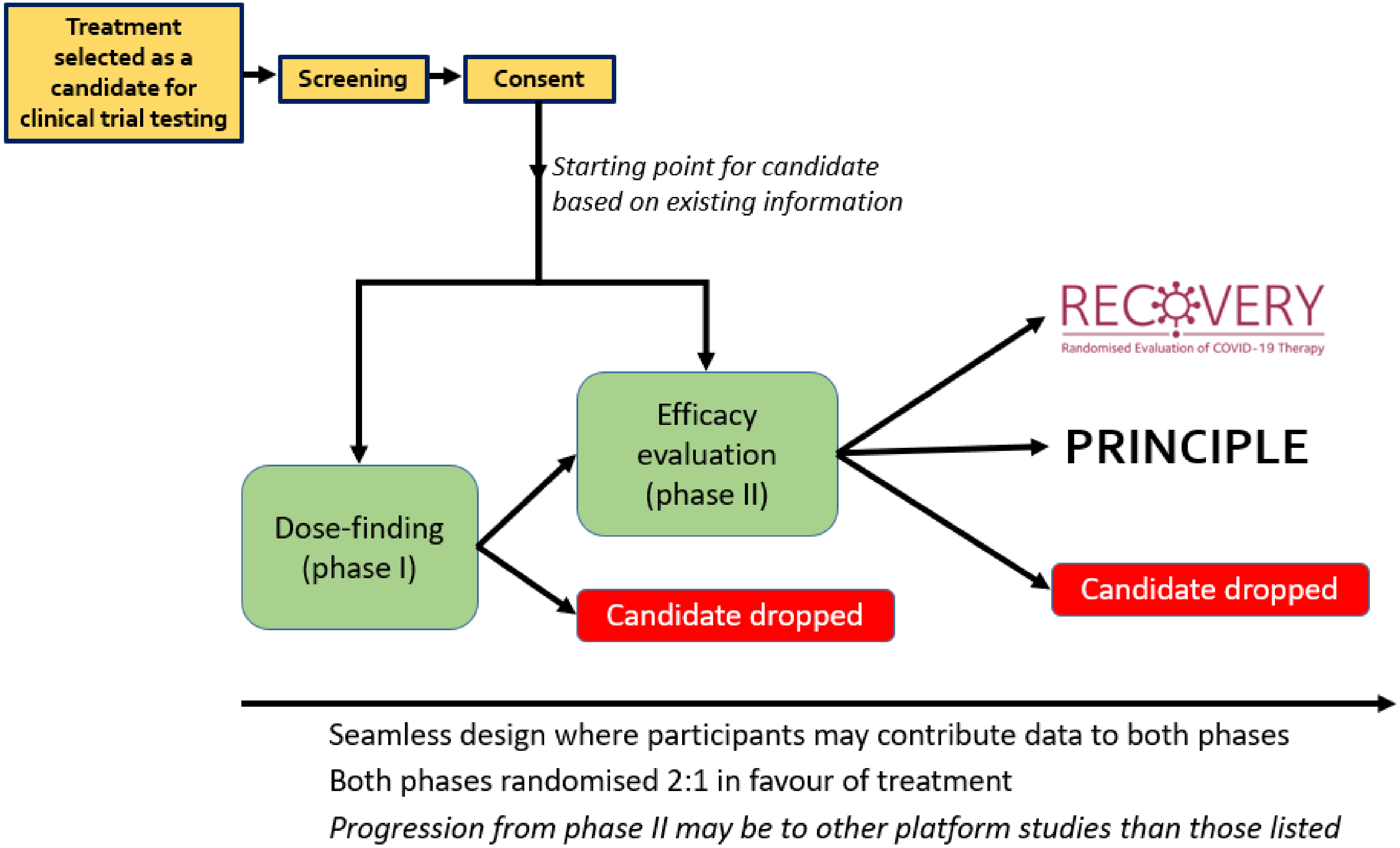

1.3 EXAMPLE SCHEDULE OF OBSERVATIONS AND PROCEDURES

Please refer to the candidate-specific trial protocol for each candidate specific schedule of observations and procedures.

<table>
<tr><th></th><th>Screening
(up to 4 days prior to randomisation)</th><th>Baseline
(Day of randomisation (Day 1))</th><th>Treatment
(to commence on day of randomisation)</th><th>Daily whilst in hospital</th><th>Specific Assessments on Days 1, 3, 5, 8, 11</th><th>Day 15
(±2 days)</th><th>Day 29
(±2 days)</th></tr>
<tr><td>Informed consent</td><td>X</td><td></td><td></td><td></td><td></td><td></td><td></td></tr>
<tr><td>SARS-Cov-2 nose/throat swab for viral titres PCR</td><td>X</td><td>X</td><td rowspan="8">Refer to CST protocol</td><td></td><td>X</td><td>X</td><td></td></tr>
<tr><td>WHO Clinical Progression Scale[8]</td><td>X</td><td>X</td><td>X</td><td></td><td>X</td><td>X</td></tr>
<tr><td>NEWS2/qSOFA Assessment and Score</td><td></td><td>X</td><td>X</td><td></td><td>X</td><td>X</td></tr>
<tr><td>Full Blood Count</td><td>X</td><td></td><td rowspan="5">Refer to CST protocol</td><td>X</td><td>X</td><td>X</td></tr>
<tr><td>U&Es</td><td>X</td><td></td><td>X</td><td>X</td><td>X</td></tr>
<tr><td>Estimated GFR</td><td>X</td><td></td><td>X</td><td>X</td><td>X</td></tr>
<tr><td>LFTs</td><td>X</td><td></td><td>X</td><td>X</td><td>X</td></tr>
<tr><td>Urinary analysis</td><td>X</td><td></td><td></td><td></td><td></td></tr>
<tr><td>Pregnancy test (urine or serum)</td><td>X</td><td colspan="6">Refer to CST protocol</td></tr>
<tr><td>Medical history (including COVID-19 history)</td><td>X</td><td></td><td rowspan="3"></td><td></td><td></td><td></td><td></td></tr>
<tr><td>Con-med/SoC review</td><td>X</td><td>X</td><td>X</td><td>X</td><td>X</td><td>X</td></tr>
<tr><td>Height (if possible)</td><td>X</td><td></td><td></td><td></td><td></td><td></td></tr>
</table>

	Screening (up to 4 days prior to randomisation)	Baseline (Day of randomisation (Day 1))	Treatment (to commence on day of randomisation)	Daily whilst in hospital	Specific Assessments on Days 1, 3, 5, 8, 11	Day 15 (±2 days)	Day 29 (±2 days)
**Weight (if possible)**	X			Refer to CST protocol			
**Assessment of oxygen use**	X	X		X			
**Assessment of mechanical ventilation use**	X	X		X			
**Physical exam**		X		As per SoC			
**Demographics**	X						
**Randomisation**		X					
**AE assessment**	X (from consent)	X		X	X	X	X
**SARS-Cov-2 nose/throat swab stored for future translational research (optional)**		X					
**Chest X-ray/other chest imaging**	If clinically indicated						
**PD/PK assessment**	Refer to CST protocol						
**Biomarker collection for translational research**	Refer to CST protocol						

2 INTRODUCTION

2.1 BACKGROUND AND RATIONALE

In 2019 a novel coronavirus-induced disease (COVID-19) emerged in Wuhan, China which was identified as a new beta-coronavirus (SARS coronavirus 2, or SARS-CoV-2)[1.] The clinical manifestations of COVID-19 range from asymptomatic infection or mild, transient symptoms to severe viral pneumonia with respiratory failure. Many patients do not progress to severe disease but as COVID-19 spreads across the UK we are seeing significant rises in the number of hospitalized pneumonia patients, and the frequency of severe disease in hospitalised patients can be as high as 30%[2,4-5]. The progression from prodrome (usually fever, fatigue and cough) to severe pneumonia requiring oxygen support or mechanical ventilation often takes one to two weeks after the onset of symptoms [2]. The kinetics of viral replication in the respiratory tract are not well characterized, but this relatively slow progression provides a potential time window in which antiviral therapies could influence the course of disease. Currently there are two approved therapeutic agents available for Covid 19: dexamethasone and remdesivir[3] . A number of COVID-19 late phase trial platforms have been developed in the UK investigating (often repurposed) drugs (e.g. RECOVERY www.recoverytrial.net), but there is an unmet need to develop early phase trial platforms to investigate novel candidates, for which promising candidates can feed these established later phase platforms over the coming months.

2.1.1 AGILE Clinical Trials Program

Given the rapid spread of COVID-19 and subsequent demands on healthcare systems, a robust but rapid assessment of potential treatments is needed. Various phase III studies have been initiated (such as SOLIDARITY, led by WHO, and RECOVERY, led by the University of Oxford) to assess repurposed treatments; these studies are designed to be flexible to allow dropping of unpromising treatments or the addition of further potential treatments. There is a need for a second and third wave of potential treatments to be evaluated alongside, in the event of the first wave of candidates should prove to be unsuccessful; this is the gap that the phase II platform trial programs aim to fill, by identifying new treatments with promising evidence of efficacy that can be further evaluated in these larger phase III trials. The AGILE trial program aims to undertake phase I/IIa that will feed into these later phase platforms.

The AGILE clinical trials program brings together expertise of the NIHR Health Protection Research Unit for Emerging and Zoonotic Diseases, NIHR Respiratory Translational Research Collaboration, NIHR Southampton Clinical Trials Unit, University of Lancaster, University of Liverpool and the NIHR Clinical Research Facility (CRF) network to enable the rapid development, conduct and reporting of clinical trials of candidate agents (Investigational Medicinal Products (IMPs)) for the treatment of COVID-19. AGILE is a seamless phase I/II (including first-in-human) study to establish the optimum dose and determine the activity and safety of each candidate and recommend whether it should be evaluated further in a later phase platforms.

The AGILE platform master protocol allows incorporation of a range of identified and yet-to-be-identified candidates as potential treatments for adults with COVID-19 into the trial. Candidates will be added into the trial via candidate-specific trial protocols of this master protocol as appendices. Having one master protocol ensures different candidates are evaluated in the same consistent manner and opening up new trials for new candidates is more efficient. Inclusion of new candidates will be determined by the independent COVID-19 Therapeutics Advisory Panel (UK-CTAP) based on pre-clinical data, evidence in the clinical setting and GMP capabilities. The UK-CTAP will specify an appropriate set of doses to be evaluated for a given candidate, dictated by existing knowledge of the candidate from any prior use in humans. A safety review committee and/or data monitoring and ethics committee (DMEC) will be responsible for deciding whether to:

1. Dose escalate (where possible);
2. Expand efficacy testing for a given dose;
3. Recommend dropping a dose or candidate from further assessment (based on futility); or

4. Recommend a dose/candidate for further testing (in a Phase II or Phase II/III trial) based on promising evidence for efficacy.

The broad design of AGILE is flexible depending on the requirement of the candidate (see Trial Schema). Where applicable, dose finding will be carried out with safety assessment (phase I), followed by a seamless assessment of efficacy (with further review of safety; phase II). The scale of these phases will depend on what information is already available on a candidate (with respect to safety) and where a candidate is most likely to progress in terms of an external Phase II or II/III study. At the end of the assessment for each candidate, a decision will be made which confirms whether the candidate is recommended to be taken into a later phase trial and, if so, at which dose. Each candidate in AGILE is evaluated individually (i.e., results of one will not impact on another), even if several candidates might undergo testing contemporaneously. We will refer to the information generated on a single candidate as coming from a candidate-specific trial (CST), which may involve any or all of the different phases described above.

2.1.2 *AGILE Dose finding (Phase I)*

The first aim of a candidate-specific trial (CST) is to estimate the dose-toxicity relationship to determine the safety profile of a candidate and inform what dose(s) is to be assessed for efficacy. Candidates may be assessed using different methods depending on what information is available to inform study design (e.g., number of doses to be evaluated), the detail of which will be included in the CST protocol. This includes the possibility that phase I of a candidate may involve two parts, Ia (healthy volunteers) and Ib (in patients). When AGILE was initiated we expected all CST phase 1s to be conducted in COVID-19 patients. However, for some phase 1 candidates enrolment of healthy volunteers is more appropriate (for example when evaluating whether supratherapeutic dosing of a repurposed drug can achieve target plasma concentrations). In these circumstances, the CST protocol will be written to allow inclusion of healthy volunteers, justifying the reasons for their inclusion.
A Bayesian model of dose toxicity, which makes dose recommendations based on anticipated toxicity rates, will be the primary analysis unless otherwise indicated (e.g., when only a safety run-in on a single dose is proposed; here, simpler analyses with stop or go criteria will be agreed in advance with oversight committees). Where possible within the design (e.g., with same eligibility criteria for phase I and II; same randomisation schedule; same care in the control arm), the candidate will be seamlessly evaluated for efficacy, where data provided in phase I (at the dose assessed in phase II) will contribute to the efficacy evaluation in phase II.

2.1.3 AGILE Efficacy (and safety) evaluation (Phase II)

The next aim of the CST is to obtain further data on efficacy (and safety) of candidates, which can be used to consider inclusion into other Phase II or II/III trials. It is anticipated that these studies may require different levels of evidence in order to consider a candidate for inclusion, and hence this phase is designed to be flexible in terms of sample size.

For any general candidate - we would only consider bypassing Ib and going straight to IIa if:

- We are evaluating a repurposed drug at its licensed dose, and there was no expectation of excess toxicity with a different indication in COVID patients
- We are evaluating a repurposed drug at a higher dose, where prior data (eg original SAD/MAD) indicated tolerability, or the drug is deemed to have an excellent safety profile
- We are evaluating a novel compound which has healthy volunteer data (SAD/MAD) outside of AGILE at the dose proposed
- We are evaluating any compound which would be included in any Phase II platforms like Recovery+ (ie already passed all checks) lacking only preliminary efficacy data – this would be done at the request of, and in close collaboration with relevant candidate prioritisation taskforces e.g. CTAP
- We would consider any compound at the request of the appropriate regulatory bodies e.g. MHRA or SAHPRA

2.2 RISK BENEFITS FOR CURRENT TRIAL

There are currently limited approved treatments for COVID-19 apart from dexamethasone and Remdesivir in hospitalised patients, and participants into AGILE will not be prevented from taking these as their Standard of Care treatment if their clinician feels they are appropriate to receive them. Although there may not be benefits for an individual entering into the trial, the existing standard of care will be maintained, and there could be a significant benefit for future COVID-19 patients should this lead to a new efficacious and safe treatment during the current global COVID-19 outbreak. For each new candidate under investigation, findings from the pre-clinical and/or any clinical trials will be briefly described with a summary of the findings described in the IB/SmPC with associated risks for that candidate in the CST protocol.

It is recognised that as evidence for efficacy accrues for new interventions, Standard of Care will change. Our statistical approaches will be adapted, including estimations for sample size, and use of controls from other CSTs which are not contemporaneous.

3 OBJECTIVES AND ENDPOINTS

Depending on the candidate being investigated the population used for evaluation may differ including only hospitalised only patients (Group A), more mild-moderate patients (Group B) or healthy volunteers (Group C). The primary endpoint will be different depending on the population used for evaluation.

Table 1 Study Objectives and Endpoints

Objectives	Endpoints
Primary	Co-primary endpoints
**Dose-finding/Phase I:**	
• To determine a dose(s) for efficacy evaluation	Dose limiting toxicities (Safety and Tolerability of drug under study – CTCAE v5 Grade ≥3 adverse events)
**Efficacy evaluation/Phase II**	
• To determine activity and safety and recommend whether a candidate should be evaluated further	In severe patients (**Group A**): time to clinical improvement. Improvement will be determined according to the WHO Clinical Progression Scale[8]; improvement is defined as a minimum 2-step change from randomisation in the scale up to day 29 post-randomisation:<br><br>WHO Clinical Progression Scale[8]:<br>0. Uninfected, no viral RNA detected<br>1. Ambulatory mild disease, asymptomatic; viral RNA detected<br>2. Ambulatory mild disease, symptomatic; independent<br>3. Ambulatory mild disease, symptomatic; assistance needed<br>4. Hospitalised moderate disease, no oxygen therapy (If hospitalised for isolation only, record status as for ambulatory patient)<br>5. Hospitalised moderate disease, oxygen by mask or nasal prongs<br>6. Hospitalised severe disease, oxygen by NIV or high flow<br>7. Hospitalised severe disease, intubation and mechanical ventilation, pO_2/FiO_2 ≥150 or SpO_2/FiO_2 ≥200<br>8. Hospitalised severe disease, mechanical ventilation pO_2/FiO_2 <150 (SpO_2/FiO_2 <200) or vasopressors<br>9. Hospitalised severe disease, mechanical ventilation pO_2/FiO_2 <150 and vasopressors, dialysis, or ECMO<br>10. Dead<br><br>In mild to moderate patients (**Group B**): pharmacodynamics of drug under study, defined as time to negative viral titres in nose and/or throat swab, measured up to 15 days post-randomisation.
Secondary	

**Phase I:**	
• Safety	• Safety: Adverse event rate according to CTCAE v5
• PD/PK (see CST)	
**Phase II:**	
• To evaluate time to, and proportion of, clinical improvement	• Proportion of patients with clinical improvement (as defined above) at day 8, 15 and day 29<br>• Change at day 8 and 15 from randomisation in the WHO Clinical Progression Scale[8]<br>• Time to a one point change on the WHO Clinical Progression Scale[8]<br>• The ratio of the oxygen saturation to fractional inspired oxygen concentration (SpO_2/FiO_2)
• To evaluate the time to, and proportion of, discharge (Group A patient trials)	• Time to discharge from randomisation<br>• Proportion of patient discharged by days 8, 15 and 29
• To evaluate admission to ICU	• Admission rate and time in ICU
• To evaluate safety further	• White cell count, haemoglobin, platelets, creatinine, and ALT on day 1, 3, 5, 8, 11 (while hospitalised); and Day 15 and 29
• To evaluate overall mortality	• Mortality at Days 8, 15 and 29<br>• Time to death from randomisation
• To evaluate the number of oxygen-free days	• Duration (days) of oxygen use and oxygen-free days
• To evaluate ventilator-free days and incidence and duration of new mechanical ventilation use	• Duration (days) of mechanical ventilation and mechanical ventilation-free days<br>• Incidence of new mechanical ventilation use and duration (days) of new mechanical ventilation use
• Treatment adherence	• Actual versus planned candidate treatment received
• To evaluate National Early Warning Score (NEWS)2/qSOFA	• NEWS2/qSOFA assessed daily while hospitalised
• To evaluate translational outcomes	• Change in viral load over time<br>• SARS-CoV-2 in OP swab at baseline<br>• Biomarkers for response

The primary outcome was chosen from a list of candidates and was deemed most suitable for a variety of reasons. The WHO ordinal scale is used in existing COVID trials such as SOLIDARITY, and is recommended by WHO; however, anecdotally, existing studies have chosen time to event outcomes rather than a fixed time point (to assess, e.g., actual score on the WHO scale) due to the high dependence on the time chosen to evaluate a treatment. Simulations by the AGILE team have shown that time to event outcomes are likely to be as efficient as using the ordinal outcome (in terms of sample size) and are more efficient than a single binary endpoint such as mortality or discharge at day 29. Although a two-point change on such a small scale might ordinarily be considered very large, emerging evidence [6] suggests that the vast majority patients have been discharged or have died by day 28, suggesting that patients end up in one end of the scale after a short period of time and so move along the scale substantially and rapidly.

4 STUDY DESIGN

4.1 OVERALL DESIGN

AGILE is a multicentre, multi-candidate, multi-dose, multi-stage, randomised phase I/II Bayesian adaptive platform trial to determine the safety and efficacy of multiple candidate agents for the treatment of COVID-19. The multi-candidate design allows many candidates to be tested simultaneously (while potentially sharing control group data, provided they are at least contemporaneously recruited), in order to increase efficiency compared to multiple single-candidate studies. The multi-dose feature allows progression beyond the licensed dose dependent on adequate safety and tolerability data, and promising efficacy. Dosing decisions may include evidence from PKPD modelling - e.g., supporting information that escalating dose is likely to increase PD effect - where this is available in the short timeframes. The multi-stage feature allows for pre-specified analyses that can be used to determine dropping of ineffective doses or candidates or recommending doses or candidates for further phase II/III testing, thus increasing efficiency of the study. The adaptive platform design allows for the removal of unpromising candidates, promising candidates to be recommended for further testing in external phase II/III studies, and the addition of new potential treatments to be added during the trial. Candidates will be added into the AGILE via candidate-specific trial (CST) protocols of this master protocol as appendices.

4.2 TRIAL PHASES

4.2.1 DOSE FINDING (PHASE I)

The first aim of a candidate-specific trials (CST) is to estimate the dose-toxicity relationship to inform which dose is to be assessed for efficacy. The details of the design will be finalised prior to first dose.

One potential difference between CSTs is the requirement for a phase Ia study in healthy volunteers. This will be assessed on a per candidate basis, as will details regarding the design, such as randomisation. Phase Ib will be randomised to the candidate or control (best supportive care). This choice is based on the still emerging nature of the symptoms associated with COVID-19 and the desire to avoid labelling potential treatments as unsafe due to misclassifying non-treatment related toxicities. Randomisation will be 2:1 in favour of treatment and contemporaneous control data from other treatment arms will be considered for inclusion in the candidate’s dose-toxicity model in order to improve the precision of the model. It is anticipated that each treatment will be evaluated in a single site during Phase I, but this will be reviewed for each CST protocol. We acknowledge the potential lack of generalisability arising from such a set-up; however, this is desirable for logistical purposes and the rapid evaluation of candidates. This is also likely to be most necessary for first-in-human studies, where there are a limited number of facilities able to conduct such studies. The borrowing of control participants from different sites will be assessed for each CST and decided in advance of recruitment to a CST; the decision will be based on the desire to avoid biases arising through the comparison of a candidate delivered in one site against control participants recruited across other sites.

Where indicated (e.g., where more than one dose is being assessed), the dose-toxicity relationship will be assessed using an adaptive Bayesian model, which will make dose recommendations based on anticipated toxicity for potential doses. Once a dose has been established as suitable for further evaluation (recommended dose for efficacy evaluation; RDEE), the candidate dose will be seamlessly evaluated for efficacy; this means that participants who have already received the RDEE during dose-finding will contribute their data to efficacy evaluation (assuming that no significant changes are required to the candidate-specific protocol prior to expansion).

Phase Ia will recruit an initial cohort of (minimum) size six before evaluation of safety and tolerability at a fixed time post initiation of treatment. Phase Ib will also recruit initial cohort of (minimum) size six will be recruited for a given treatment (randomised as 4 to candidate and 2 to control). Treatment will be evaluated for safety and tolerability at a fixed time post-randomisation. All accrued information will inform dose escalation decisions. If it is deemed that a higher dose is likely to be sufficiently safe, then a new cohort of the same size will be recruited for the next, higher dose (as specified in CST protocol). This decision-making may be supported by PKPD modelling; however, it is not anticipated that this will generally be available given the short timeframes in this phase. The number of doses to be investigated in this trial is flexible, although the best trade-off between number of patients required and power is 3-4 doses. Once

the highest safe dose/maximum tolerated in order to maximise potential therapeutic effect, has been established, seamless expansion to phase II will occur and all participants treated at the RDEE during dose-finding will be included in the phase II efficacy evaluation. Safety and tolerability for a given treatment will be based on comparisons to the control group and potentially other contemporaneously recruited control participants to avoid any potential bias arising from the case mix changing over time.

Full details on each study specifics are outlined in CST protocols.

4.2.2 EFFICACY EVALUATION (PHASE II)

Phase II will be randomised in the same manner as phase I (2:1 in favour of the candidate). Participants treated with the RDEE during dose-finding will contribute data to the efficacy evaluation. Similarly, additional patients recruited for efficacy evaluation will also contribute to the refinement of the dose-toxicity model. The efficacy of a candidate will be established through comparison to control, potentially supplemented by contemporaneously recruited controls from other CSTs (expected to be no more than 20 in the majority of candidates). As new candidates will be ready for efficacy evaluation at different times, direct comparisons between candidates is unlikely to be possible; hence, screening designs such as those proposed by Simon et al.[7] are not feasible.

Potential for efficacy will be assessed using the posterior probability that the hazard ratio is greater than one. Three actions are possible following efficacy evaluation:

- Stop further evaluation of a dose or candidate due to the probability of having a promising effect being too low;
- Recommend opening an additional cohort of (at least) six participants (with same allocation ratio) at the same dose; or
- Recommend that dose to be recommended for further evaluation in a late phase trial platform.

The design has been constructed so that, in each pair-wise comparison of the candidate and control, the probability of concluding that a dose is efficacious when it is not, is no larger than 10%. The power to detect an efficacious dose is 60% under a hazard ratio of 1.75 (which under the made assumptions implies a reduction in the median time to improvement in Group A from 14 to 8 days or that the time to negative viral titers is reduced from 14 to 8 days in Group B). The size of the expansion of phase II may depend on external factors such as which follow-on study the treatment is likely to be assessed in, whereby different levels of evidence are required to support the rationale for a given treatment to be taken on in another platform study. Rules for expansion will be specified in the CST protocol.

The safety review committee (SRC) and/or data monitoring and ethics committee (DMEC) will evaluate safety and efficacy or futility of each candidate (details below). If, during the course of the study, standard care changes, this will become the new control group for all future treatments and comparisons. The DMEC will be asked to review the use of candidates currently undergoing assessment to decide how to best use existing information on the candidates.

Stopping criteria for harm or futility

Assessment of a candidate for safety will cease if the probability that the risk of toxicity is 30% more than the control arm is 25% or more. Harm is defined by unacceptable toxicity as given by the Common Terminology Criteria for Adverse Events (CTCAE) criteria (v5.0 grade ≥3):

- Grade 3 Severe or medically significant but not immediately life-threatening; hospitalization or prolongation of hospitalization indicated; disabling; limiting self-care ADL
- Grade 4 Life-threatening consequences; urgent intervention indicated
- Grade 5 Death

Futility will be defined by:

- Probability that hazard ratio of time to negative viral titers (Group B) or clinical improvement (Group A) is >1 is less than 33% (i.e., evidence of low chance of efficacious treatment).

Stopping criteria for efficacy
Efficacy will be determined if the probability of the hazard ratio of time to negative viral titers (Group B) or clinical improvement (Group A) being larger than 1 is greater than 80% (i.e., evidence of efficacy).

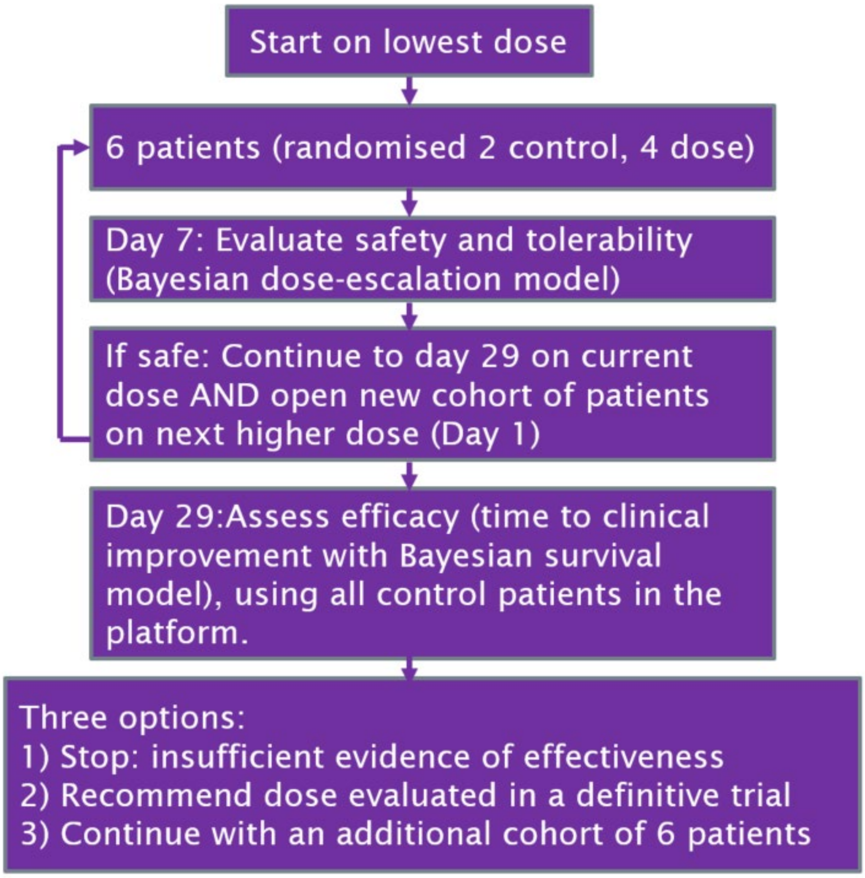

Figure 1: Trial schema for dose finding and efficacy evaluation
NB: Trials can also be stopped for safety

4.3 JUSTIFICATION FOR DOSE

Justification for dose of each candidate will be included in each CST protocol.

4.4 DEFINITION OF END OF TRIAL

For regulatory purposes the end of the trial will be when the last participant in the last CST protocol has completed the last data point.

Once the end of trial has been declared, no more prospective participant data will be collected but sites must co-operate with any data queries regarding existing data to allow for analysis and publication of results.

5 SELECTION AND ENROLMENT OF PARTICIPANTS

5.1 INFORMED CONSENT

Informed consent is a process that is initiated prior to an individual agreeing to participate in a trial and continues throughout the individual's participation. In obtaining and documenting informed consent, the investigator should comply with applicable regulatory requirements and should adhere to the principles of GCP.

Discussion of objectives, risks and inconveniences of the trial and the conditions under which it is to be conducted are to be provided to the participant, where possible, by appropriately delegated staff from the research team with knowledge in obtaining informed consent with reference to the participant information sheet. This information will emphasise that participation in the trial is voluntary and that the participant may choose not to take part or withdraw from the trial at any time and for any reason without affecting the

standard clinical care that the participant receives for treatment of COVID-19 (where applicable). The participant or delegate signing consent on behalf of the participant, will be given the opportunity to ask any questions that may arise and provided the opportunity to discuss the trial with family members, friend (if possible given the COVID-19 hospital restrictions) or an independent healthcare professional outside of the research team and time to consider the information prior to agreeing to participate.

Consent to enter the trial must be sought, where possible, from each participant only after a full explanation has been given, a Participant Information Sheet offered (either a paper copy or on a tablet) and appropriate time allowed for consideration.

If the patient lacks capacity to give consent due to the severity of their medical condition (e.g. patients with WHO Clinical Progression Scale[8] of 7-9 (hospitalised, severe disease), then in the first instance, consent may be obtained from the patient's personal legal representative. Patient's in hospital with COVID-19 are treated in 'red areas'. These areas are restricted and visitors (i.e. relatives/friends) are not usually permitted to visit. In these situations, the research team should take all reasonable steps to identify a personal legal representative to discuss the trial over the phone. The phone call will be followed up with the Legal Representative Information Sheet being emailed (or posted if sufficient time) to the personal legal representative to read. After being given sufficient time to ask questions and consider their relative's/friend's participation in the trial, a clinician independent of the research team will have a telephone call with the personal legal representative to receive the personal legal representative's opinion. Following the phone call, the independent clinician will document the call in the patient's notes and, if appropriate, sign the Informed Consent Form for legal representatives. In situations where the personal legal representative is present in person at the hospital, the Personal Legal Representative Informed Consent Form will be used.

If the research team is unable to contact a personal legal representative, informed consent can be provided by a treating clinician (independent of the clinician seeking to enrol the patient) who will act as the professional legal representative. Following this, the Next of Kin Letter together with the Legal Representative Information Sheet will be sent to the patient's next of kin (if one exists) as soon as possible to inform them of the decision taken and provide further information, including contacts details for further discussions.

Further informed consent will then be sought with the patient if they recover sufficiently using the Recovered Capacity Participant Information Sheet and Informed Consent Form.

All forms of consent will be received and signed by a delegated member of the research team.

The right of the participant/legal representative to refuse to participate without giving reasons must be respected. After the participant has entered the trial the clinician remains free to give alternative treatment to that specified in the protocol at any stage if he/she feels it is in the participant's best interest, but the reasons for doing so should be recorded. In these cases the participants remain within the trial for the purposes of follow-up and data analysis. All participants/legal representatives are free to withdraw at any time from the protocol treatment without giving reasons and without prejudicing further treatment.

If a participant is able to consent for the trial but later becomes incapacitated, the original consent will endure for the loss of capacity.

Note: *The AGILE platform was developed in the UK, where it has received ethics approval from the Human Research Authority (HRA) to recruit participants incapable of consent (such as unconscious patients in intensive care). This is justified on the basis that some candidate interventions will be targeted at this group of sickest patients for whom mortality still remains high (over 30%); evaluation of early phase efficacy could not be meaningfully carried out in any other group. The UK has established ethical practice, and strong protections for undertaking CTIMPs in such patients. This includes the requirement to seek independent medical consent, and only after all other avenues of gaining consent (with respect to next-of-kin) have been exhausted. Moreover, to go straight into phase IIb/III trials after evaluation only in healthy volunteers places larger numbers of people at risk compared to the small, closely monitored trials that are proposed. We have therefore taken the view (and the HRA has agreed with this view) that in these circumstances, and under these protections, the benefits outweigh the risks.*

We recognise that for AGILE sites outside of the UK, local guidelines and established practice may differ. Where this is the case, while the minimum ethical standards of the UK apply, we will always defer to any additional ethical constraints, and will not recruit unconscious patients without consent where this is not deemed ethically acceptable.

5.2 INCLUSION CRITERIA

N.B. The CST protocol inclusion criteria will take precedence over the master protocol inclusion criteria.

Patients are eligible to be included in the study only if all of the following criteria apply (as well as all criteria from the appropriate CST protocol):

1. Adults (≥18 years) and in Groups A and B, with laboratory-confirmed* SARS-CoV-2 infection (PCR)
2. Ability to provide informed consent signed by study patient or legally acceptable representative
3. Women of childbearing potential (WOCBP, as defined in section 5.5 below) and male patients who are sexually active with WOCBP must agree to use a highly effective method of contraception (as outlined in section 5.6 below) from the first administration of trial treatment, throughout trial treatment and for the duration outlined in the candidate-specific trial protocol after the last dose of trial treatment.

*If any CSTs are included in the community setting, the CST protocol will clarify whether patients with suspected SARS-CoV-2 infection are also eligible.

Standard additional criteria that may be applied per CST protocol:

Group A (severe disease)

4a. Patients with clinical status of Grades 5 (hospitalised, oxygen by mask or nasal prongs), 5 (hospitalised, on non-invasive ventilation, or high flow oxygen), 7 (hospitalised, intubation and mechanical ventilation, pO_2/FiO_2 ≥150 or SpO_2/FiO_2 ≥200), 8 (hospitalised mechanical ventilation pO_2/FiO_2 <150 (SpO_2/FiO_2 <200) or vasopressors) or 9 (hospitalised, mechanical ventilation pO_2/FiO_2 <150 and vasopressors, dialysis or ECMO), as defined by the WHO Clinical Progression Scale[8].

Group B (mild-moderate disease)

4b. Ambulant or hospitalised patients with the following characteristics peripheral capillary oxygen saturation (SpO_2) >94% RA

Group C (Healthy Volunteers)

4c. To be defined in CST protocols

5.3 EXCLUSION CRITERIA

Patients are excluded from the study if any of the following criteria apply (as well as all criteria from the appropriate CST protocol):

1. Alanine aminotransferase (ALT) and/or aspartate aminotransferase (AST) >5 times the upper limit of normal (ULN)
2. Stage 4 severe chronic kidney disease or requiring dialysis (i.e., estimated glomerular filtration rate <30 mL/min/1.73 m^2)
3. Pregnant or breast feeding
4. Anticipated transfer to another hospital which is not a study site within 72 hours
5. Allergy to any study medication
6. Patients taking other prohibited drugs (as outline in CST protocol) within 30 days or 5 times the half-life (whichever is longer) of enrolment
7. Patients participating in another CTIMP trial (unless specifically stated in the CST)

N.B. The CST protocol exclusion criteria will take precedence over the master protocol exclusion criteria.

5.4 SCREEN FAILURES

Screen failures are defined as patients who consent to participate in the clinical study but are not subsequently assigned to study treatment. A minimal set of screen failure information is required to ensure transparent reporting of screen failure patients to meet the Consolidated Standards of Reporting Trials (CONSORT) publishing requirements and to respond to queries from regulatory authorities. Minimal information includes demography and screen failure details.

Individuals who do not meet the criteria for participation in this study (screen failure) may be rescreened.

5.5 CONTRACEPTION

Definitions of women of childbearing potential (WOCBP) and fertile men:

A woman of childbearing potential (WOCBP) is a sexually mature woman (i.e. any female who has experienced menstrual bleeding) who has not:

- Undergone a hysterectomy or bilateral oophorectomy/salpingectomy
- Been postmenopausal for 12 consecutive months (i.e. who has not had menses at any time in the preceding 12 consecutive months without an alternative medical cause)
- Had premature ovarian failure confirmed by a specialist gynaecologist

A man is considered fertile after puberty unless permanently sterile by bilateral orchiectomy.

Female Patients

To be considered eligible for the trial, all female patients who are WOCBP must consent to use one of the following methods of highly effective contraception from the first administration of study treatment, throughout trial treatment and for the duration outlined in the candidate-specific trial protocol after the last dose of trial treatment:

- Combined (estrogen and progestogen containing) hormonal contraception associated with inhibition of ovulation[A]:
    a. Oral
- Intravaginal
- Transdermal
- Progestogen-only hormonal contraception associated with inhibition of ovulation[1]:
    a. Oral
    b. Injectable
    c. Implantable[B]
- Intrauterine device (IUD)[B]
- Intrauterine hormone-releasing system (IUS)[B]
- Bilateral tubal occlusion[B]
- Vasectomised partner[B,C]
- Sexual abstinence[D]

[A]Hormonal contraception may be susceptible to interaction with the IMP, which may reduce the efficacy of the contraception method.

[B]Contraception methods that in the context of this guidance are considered to have low user dependency.

[C]Vasectomised partner is a highly effective birth control method provided that partner is the sole sexual partner of the WOCBP trial participant and that the vasectomised partner has received medical assessment of the surgical success.

[D]In the context of this guidance sexual abstinence is considered a highly effective method only if defined as refraining from heterosexual intercourse during the entire period of risk associated with the study

treatments. The reliability of sexual abstinence needs to be evaluated in relation to the duration of the clinical trial and the preferred and usual lifestyle of the participant.

Male patients
To be considered eligible for the trial, male patients (including those with partners of child-bearing potential) must consent to use the following methods of contraception from the first administration of study treatment, throughout trial treatment and for duration outlined in the candidate-specific trial protocol after the last dose of trial treatment:

1. Condom
2. Female partner to use one of the highly effective methods of contraception detailed above

Male patients must also refrain from donating sperm during this period.

5.6 REGISTRATION / RANDOMISATION PROCEDURES

To be outlined in each candidate specific protocol. For healthy volunteer studies this may also include cohort management procedures.

6 STUDY TREATMENT

Please reference to the CST protocols for details on treatment schedule, IMP (candidate) supply, administration, accountability, concomitant medications, prohibited and restricted therapies during the trial and dose delays and modifications.

Each dose of candidate agent will be administered by a member of the clinical research team, that is qualified and licensed to administer the study product. Administration and date, time, and location of injection (if relevant) will be entered into the eCRF which will be used to oversee drug accountability and determine treatment compliance.

SoC should be based on appropriate guidelines in place at the time of treatment on the study, for example the current National Institute for Health and Care Excellence 'COVID-19 rapid guideline: critical care in adults'.

7 DISCONTINUATION OF STUDY TREATMENT AND PATIENT DISCONTINUATION/WITHDRAWAL

7.1 TRIAL DISCONTINUATION

In consenting to the trial, participants have consented to the trial intervention, follow-up and data collection. Participants may be discontinued from the trial procedures at any time.

Participants may be discontinued from the trial in the event of:

- Clinical decision, as judged by the Principal Investigator or CI
- Pregnancy (refer to Safety section below for follow up requirements)
- Termination of trial by sponsor
- Participant choice

Full details of the reason for trial discontinuation should be recorded in the eCRF and medical record.

7.2 WITHDRAWAL

The participant/legal representative is free to withdraw consent from the trial at any time without providing a reason.

Investigators should explain to participants the value of remaining in trial follow-up and allowing this data to be used for trial purposes. Where possible, participants who have withdrawn from trial treatment should remain in follow-up as per the trial schedule. If participants additionally withdraw consent for this, they should revert to standard clinical care as deemed by the responsible clinician. It would remain useful for the

trial team to continue to collect standard follow-up data and unless the participant explicitly states otherwise, follow-up data will continue to be collected.

Details of trial discontinuation (date, reason if known) should be recorded in the eCRF and medical record.

The strategy for dropouts/withdrawals should be defined in individual CST protocols due to different requirements depending on stage of trial eg. First in human vs Phase IIa.

7.3 LOST TO FOLLOW UP

A participant will be considered lost to follow-up if they cannot be contacted after discharge from the hospital.

Before a participant is deemed lost to follow-up, the site must make every effort to regain contact with the participant and to confirm whether the participant is alive or has died post hospital discharge. These contact attempts should be documented in the participant's medical record.

8 STUDY ASSESSMENTS AND PROCEDURES

8.1 SCREENING PROCEDURES

Screening procedures to be carried out up to 4 days prior to randomisation for all CSTs will include the following. Refer to CST protocols for additional screening procedures:

- Informed consent
- SARS-CoV-2 nose/throat swabs for viral titres PCR (if not already performed within the 4 days prior to screening)
- Assessment using the WHO Clinical Progression Scale[8]:
  0. Uninfected, no viral RNA detected
  1. Ambulatory mild disease, asymptomatic; viral RNA detected
  2. Ambulatory mild disease, symptomatic; independent
  3. Ambulatory mild disease, symptomatic; assistance needed
  4. Hospitalised moderate disease, no oxygen therapy (If hospitalised for isolation only, record status as for ambulatory patient)
  5. Hospitalised moderate disease, oxygen by mask or nasal prongs
  6. Hospitalised severe disease, oxygen by NIV or high flow
  7. Hospitalised severe disease, intubation and mechanical ventilation $pO_2/FiO_2 \geq 150$ or $SpO_2/FiO_2 \geq 200$
  8. Hospitalised severe disease, mechanical ventilation $pO_2/FiO_2 <150$ ($SpO_2/FiO_2 <200$) or vasopressors
  9. Hospitalised severe disease, mechanical ventilation $pO_2/FiO_2 <150$ and vasopressors, dialysis, or ECMO
  10. Dead

- Full blood count
- Urea and electrolytes
- Estimated GFR
- Liver Function Tests
- Urinary Analysis
- Women of childbearing potential: Pregnancy test (serum or urine)
- Medical history (including COVID-19 history e.g. symptom onset)
- Concomitant medication and standard of care review
- Height and weight, if possible
- Assessment of oxygen use

- Assessment of mechanical ventilation use
- Demographics review
- Adverse Event Assessment (from consent)

8.2 TRIAL PROCEDURES

Trial specific procedures may vary dependent on the candidate being used in the CST. The sections below outline the minimal assessments for Groups A and B. Please refer to the CST protocols for a full list of assessments for each CST.

Assessments and visit schedule for Group C (healthy volunteers) will differ and will be detailed in the CST protocol.

8.2.1 Baseline (i.e. Day 1, day of Randomisation)

The following assessments are to be carried out on the day of randomisation for all CSTs . Refer to CST protocols for additional procedures. **Patients should commence treatment on the day of randomisation (i.e. Day 1) if randomised to the treatment arm**.

- SARS-CoV-2 nose/throat swabs for viral titres PCR/Ag testing
- Assessment using the WHO Clinical Progression Scale[8]:
  0. Uninfected, no viral RNA detected
  1. Ambulatory mild disease, asymptomatic; viral RNA detected
  2. Ambulatory mild disease, symptomatic; independent
  3. Ambulatory mild disease, symptomatic; assistance needed
  4. Hospitalised moderate disease, no oxygen therapy (If hospitalised for isolation only, record status as for ambulatory patient)
  5. Hospitalised moderate disease, oxygen by mask or nasal prongs
  6. Hospitalised severe disease, oxygen by NIV or high flow
  7. Hospitalised severe disease, intubation and mechanical ventilation $pO_2/FiO_2 \geq 150$ or $SpO_2/FiO_2 \geq 200$
  8. Hospitalised severe disease, mechanical ventilation $pO_2/FiO_2 < 150$ ($SpO_2/FiO_2 < 200$) or vasopressors
  9. Hospitalised severe disease, mechanical ventilation $pO_2/FiO_2 < 150$ and vasopressors, dialysis, or ECMO
  10. Dead
- Non-Ventilated Patients: National Early Warning Score 2 (NEWS2) Assessment:
  - Respiration rate
  - Oxygen saturation
  - Systolic blood pressure
  - Pulse rate
  - Level of consciousness or new confusion*
  - Temperature

    *The patient has new-onset confusion, disorientation and/or agitation, where previously their mental state was normal – this may be subtle. The patient may respond to questions coherently, but there is some confusion, disorientation and/or agitation. This would score 3 or 4 on the GCS (rather than the normal 5 for verbal response), and scores 3 on the NEWS system.
- Ventilated Patients: Quick Sepsis-Related Organ Dysfunction Assessment (qSOFA) Score:
  - Blood pressure
  - Respiratory rate
  - Glasgow Coma Scale
- Concomitant medication and standard of care review
- Assessment of oxygen use

- Assessment of mechanical ventilation use
- Physical examination
- Adverse event assessment
- SARS-CoV-2 nose/throat swab for storage for future translational research

8.2.2 Treatment

Refer to CST protocols for assessments during treatment.

8.2.3 Daily Whilst in Hospital

The following assessments are to be carried out daily whilst patients are in hospital. Refer to CST protocols for additional procedures.

- Assessment using the WHO Clinical Progression Scale[8]:
  0. Uninfected, no viral RNA detected
  1. Ambulatory mild disease, asymptomatic; viral RNA detected
  2. Ambulatory mild disease, symptomatic; independent
  3. Ambulatory mild disease, symptomatic; assistance needed
  4. Hospitalised moderate disease, no oxygen therapy (If hospitalised for isolation only, record status as for ambulatory patient)
  5. Hospitalised moderate disease, oxygen by mask or nasal prongs
  6. Hospitalised severe disease, oxygen by NIV or high flow
  7. Hospitalised severe disease, intubation and mechanical ventilation $pO_2/FiO_2 \geq 150$ or $SpO_2/FiO_2 \geq 200$
  8. Hospitalised severe disease, mechanical ventilation $pO_2/FiO_2 < 150$ ($SpO_2/FiO_2 < 200$) or vasopressors
  9. Hospitalised severe disease, mechanical ventilation $pO_2/FiO_2 < 150$ and vasopressors, dialysis, or ECMO
  10. Dead
- Non-Ventilated Patients: National Early Warning Score 2 (NEWS2) Assessment:
  - Respiration rate
  - Oxygen saturation
  - Systolic blood pressure
  - Pulse rate
  - Level of consciousness or new confusion*
  - Temperature

    *The patient has new-onset confusion, disorientation and/or agitation, where previously their mental state was normal – this may be subtle. The patient may respond to questions coherently, but there is some confusion, disorientation and/or agitation. This would score 3 or 4 on the GCS (rather than the normal 5 for verbal response), and scores 3 on the NEWS system.
- Ventilated Patients: Quick Sepsis-Related Organ Dysfunction Assessment (qSOFA) Score:
  - Blood pressure
  - Respiratory rate
  - Glasgow Coma Scale
- Concomitant medication and standard of care review
- Assessment of oxygen use
- Assessment of mechanical ventilation use
- Adverse event assessment
- Physical exams as per standard of care

8.2.4 Specific Assessments on Days 1, 3, 5, 8 and 11

The following assessments are to be carried out on Days 1, 3, 5, 8 and 11 (NB day of randomisation and start date of treatment is Day 1). Refer to CST protocols for additional procedures.

- SARS-CoV-2 nose/throat swab for viral titres PCR
- Full blood count
- Urea and electrolytes
- Estimated GFR
- Liver Function Tests
- Concomitant medication and standard of care review
- AE assessment

8.2.5 Day 15 (±2 Days)

The following assessments are to be carried out on Day 15 (±2 days) (NB day of randomisation and start date of treatment is Day 1). Refer to CST protocols for additional procedures. If patients are discharged, it is anticipated that patients may return to hospital (at hospital choice based on COVID-19 restrictions on hospital access) for follow-up and blood samples If hospitals are not able to do this, follow-up should be done by phone call and a minimised criteria will apply.

- SARS-CoV-2 nose/throat swab for viral titres PCR
- Assessment using the WHO Clinical Progression Scale[8]:
  0. Uninfected, no viral RNA detected
  1. Ambulatory mild disease, asymptomatic; viral RNA detected
  2. Ambulatory mild disease, symptomatic; independent
  3. Ambulatory mild disease, symptomatic; assistance needed
  4. Hospitalised moderate disease, no oxygen therapy (If hospitalised for isolation only, record status as for ambulatory patient)
  5. Hospitalised moderate disease, oxygen by mask or nasal prongs
  6. Hospitalised severe disease, oxygen by NIV or high flow
  7. Hospitalised severe disease, intubation and mechanical ventilation $pO_2/FiO_2 \geq 150$ or $SpO_2/FiO_2 \geq 200$
  8. Hospitalised severe disease, mechanical ventilation $pO_2/FiO_2 < 150$ ($SpO_2/FiO_2 < 200$) or vasopressors
  9. Hospitalised severe disease, mechanical ventilation $pO_2/FiO_2 < 150$ and vasopressors, dialysis, or ECMO
  10. Dead
- Non-Ventilated Patients: National Early Warning Score 2 (NEWS2) Assessment:
  - Respiration rate
  - Oxygen saturation
  - Systolic blood pressure
  - Pulse rate
  - Level of consciousness or new confusion*
  - Temperature

    *The patient has new-onset confusion, disorientation and/or agitation, where previously their mental state was normal – this may be subtle. The patient may respond to questions coherently, but there is some confusion, disorientation and/or agitation. This would score 3 or 4 on the GCS (rather than the normal 5 for verbal response), and scores 3 on the NEWS system.
- Ventilated Patients: Quick Sepsis-Related Organ Dysfunction Assessment (qSOFA) Score:
  - Blood pressure
  - Respiratory rate
  - Glasgow Coma Scale
- Full blood count
- Urea and electrolytes
- Estimated GFR
- Liver Function Tests

- Concomitant medication and standard of care review
- AE assessment

8.2.6 *Day 29 (±2 Days)*

The following assessments are to be carried out on Day 29 (±2 days) (NB day of randomisation and start date of treatment is Day 1). Refer to CST protocols for additional procedures. If patients are discharged, it is anticipated that patients may return to hospital (at hospital choice based on COVID-19 restrictions on hospital access) for follow-up and blood samples If hospitals are not able to do this, follow-up should be done by phone call and a minimised criteria will apply.

- Assessment using the WHO Clinical Progression Scale[8]:
  0. Uninfected, no viral RNA detected
  1. Ambulatory mild disease, asymptomatic; viral RNA detected
  2. Ambulatory mild disease, symptomatic; independent
  3. Ambulatory mild disease, symptomatic; assistance needed
  4. Hospitalised moderate disease, no oxygen therapy
  5. Hospitalised moderate disease, oxygen by mask or nasal prongs
  6. Hospitalised severe disease, oxygen by NIV or high flow
  7. Hospitalised severe disease, intubation and mechanical ventilation $pO_2/FiO_2 \geq 150$ or $SpO_2/FiO_2 \geq 200$
  8. Hospitalised severe disease, mechanical ventilation $pO_2/FiO_2 < 150$ ($SpO_2/FiO_2 < 200$) or vasopressors
  9. Hospitalised severe disease, mechanical ventilation $pO_2/FiO_2 < 150$ and vasopressors, dialysis, or ECMO
  10. Dead
- Non-Ventilated Patients (if in hospital): National Early Warning Score 2 (NEWS2) Assessment:
  - Respiration rate
  - Oxygen saturation
  - Systolic blood pressure
  - Pulse rate
  - Level of consciousness or new confusion*
  - Temperature

    *The patient has new-onset confusion, disorientation and/or agitation, where previously their mental state was normal – this may be subtle. The patient may respond to questions coherently, but there is some confusion, disorientation and/or agitation. This would score 3 or 4 on the GCS (rather than the normal 5 for verbal response), and scores 3 on the NEWS system.
- Ventilated Patients: Quick Sepsis-Related Organ Dysfunction Assessment (qSOFA) Score:
  - Blood pressure
  - Respiratory rate
  - Glasgow Coma Scale
- Full blood count
- Urea and electrolytes
- Estimated GFR
- Liver Function Tests
- Concomitant medication and standard of care review
- AE assessment

8.3 PHARMACOKINETICS AND PHARMACODYNAMICS

Any pharmacokinetic (PK) and pharmacodynamic (PD) assessments performed will be specific to the candidate agent and will be discussed in the corresponding candidate-specific trial protocol, including a schedule for collection of samples of blood or other biological samples for analysis.

It is anticipated that, for all drugs under study, a full PK-PD profile will be undertaken at steady state. Sparse PK and PD samples will be taken at other timepoints in addition. Viral swabs will be performed at days 1, 3, 5, 8, 11 and 15. Furthermore, safety laboratory samples, vital signs and 12-lead ECGs will be obtained at timepoints defined by the known PK, PD and safety data on the drug under study. For all study drugs, baseline safety and pre-dose PK/PD samples will be obtained.

A maximum of 400mL of blood over 15 days will be taken from participants as part of the study. This is lower than the average UK blood donation. In order to ensure that blood volumes do not exceed this, PK and PD sampling will be reduced rather than sampling for safety assessments.

8.4 DEVIATIONS AND SERIOUS BREACHES

Any trial protocol deviations/violations and breaches of Good Clinical Practice occurring at sites should be reported to the SCTU and the local R&D Office immediately. SCTU will then advise of and/or undertake any corrective and preventative actions as required.

All serious protocol deviations/violations and serious breaches of Good Clinical Practice and/or the trial protocol will immediately be reported to the regulatory authorities and other organisations, as required in the Medicines for Human Use (Clinical Trials) Regulations 2004, as amended.

9 SAFETY

9.1 DEFINITIONS

The Medicines for Human Use (Clinical Trials) Regulations 2004, as amended, provides the following definitions relating to adverse events in trials with an investigational medicinal product:

Adverse Event (AE): any untoward medical occurrence in a participant or clinical trial participant administered a medicinal product and which does not necessarily have a causal relationship with this treatment.
An AE can therefore be any unfavourable and unintended sign (including an abnormal laboratory finding), symptom, or disease temporally associated with the use of an investigational medicinal product (IMP), whether or not considered related to the IMP.

Adverse Reaction (AR): all untoward and unintended responses to an IMP related to any dose administered.
All AEs judged by either the reporting investigator or the sponsor as having reasonable causal relationship to a medicinal product qualify as adverse reactions. The expression reasonable causal relationship means to convey in general that there is evidence or argument to suggest a causal relationship.

Unexpected Adverse Reaction: an AR, the nature or severity of which is not consistent with the applicable product information (e.g. investigator's brochure (IB) for an unapproved investigational product, or summary of product characteristics (SmPC) for an authorised product).
When the outcome of the adverse reaction is not consistent with the applicable product information this adverse reaction should be considered as unexpected. Side effects documented in the IB/SmPC which occur in a more severe form than anticipated are also considered to be unexpected. Reports which add significant information on specificity or severity of a known documented adverse event are to be considered unexpected.

Serious Adverse Event (SAE) or **Serious Adverse Reaction (SAR) or Suspected Unexpected Serious Adverse Reaction (SUSAR):** any untoward medical occurrence or effect that at any dose:

- **Results in death**
- **Is life-threatening*** – *refers to an event in which the participant was at risk of death at the time of the event; it does not refer to an event which hypothetically might have caused death if it were more severe*
- **Requires hospitalisation****, or prolongation of existing hospitalisation**
- **Results in persistent or significant disability or incapacity**
- **Is a congenital anomaly or birth defect**
- Important medical events***.

*‘life-threatening’ in the definition of ‘serious’ refers to an event in which the patient was at risk of death at the time of the event; it does not refer to an event which hypothetically might have caused death if it were more severe.
** Hospitalisation is defined as an inpatient admission, regardless of length of stay, even if the hospitalisation is a precautionary measure for continued observation. Hospitalisations for a pre-existing condition, including elective procedures that have not worsened, do not constitute an SAE.
***Other important medical events may also be considered serious if they jeopardise the participant or require an intervention to prevent one of the above consequences.

Note: *It is the responsibility of the PI or delegate to grade an event as ‘not serious’ (AE) or ‘serious’ (SAE).*

Suspected Unexpected Serious Adverse Reaction (SUSAR): any suspected adverse reaction related to an IMP that is both unexpected and serious.

9.2 REPORTING WINDOWS

AEs/SAEs should be reported from consent until the CST protocol specified cut off period. Please see specific CST protocols for further details.

SAE reporting for CSTs undertaken outside of the AGILE UK framework will be undertaken in accordance with national regulatory requirements, and additional procedures will be detailed in the CST.

9.3 ADVERSE EVENT TERM AND SEVERITY GRADE

An adverse event term must be provided for each adverse event. Wherever possible a valid term listed in the Common Terminology Criteria for Adverse Events (CTCAE) v5.0 should be used. This is available online at:
https://ctep.cancer.gov/protocoldevelopment/electronic_applications/docs/CTCAE_v5_Quick_Reference_5x7.pdf
Severity grade of each adverse event must be determined by using the unique clinical descriptions of severity for each AE in CTCAE v5.0.

All adverse events should be recorded in the relevant eCRF and submitted to SCTU. Each time there is a change in grade of an adverse event this should be recorded on a separate log line on the adverse event form on the eCRF. Refer to eCRF guidance for further information on how to record these.

To ensure that the SCTU can comply with requirements of onward reporting, the CTCAE terms entered by the site will be coded to MedDRA. The MedDRA hierarchies (System Organ Class etc.) associated with the terms will be utilised for reports where required.

9.4 SERIOUSNESS

A complete assessment of the seriousness must always be assessed by a medically qualified doctor who is registered on the delegation of responsibility log; this is usually the investigator.

All adverse events that fulfil the criteria definition of ‘serious’ in protocol section 9.1, must be reported to SCTU using the Serious Adverse Event Report Form (see section 9.7 below). Specific exceptions to this (as listed below) should be recorded as AEs rather than SAEs.

All SAEs must be reported immediately by the PI or delegate at the participating centre to the SCTU.

9.4.1 Exceptions:

For the purposes of this trial, the following SAEs **do not** require reporting to SCTU using the Serious Adverse Event Report Form:

- Death due to disease progression of COVID-19 – This is the condition for which the participant is being treated. Unless death is considered related to the candidate.

- Hospitalisations for elective treatment of a pre-existing condition (the pre-existing condition to be captured within the medical history CRF).
- Any other as appropriate e.g. SAEs occurring prior to trial treatment/intervention, that are not considered to be related to trial procedures
- SAEs occurring prior to the first dose of the candidate, that are not considered to be related to trial procedures

9.5 CAUSALITY

A complete assessment of the causality must always be made by a medically qualified doctor who is registered on the delegation of responsibility log; this is usually the investigator.

If any doubt about the causality exists the local investigator should inform SCTU who will notify the Chief Investigator. Other clinicians may be asked for advice in these cases.

In the case of discrepant views on causality, SCTU will classify the event as per the worst case classification.

Relationship	Denoted
**Related -** There is clear evidence to suggest a causal relationship and other possible contributing factors can be ruled out.	SAR/SUSAR
**Unrelated -** There is no evidence of any causal relationship	SAE

9.6 EXPECTEDNESS - SEE CANDIDATE-SPECIFIC TRIAL PROTOCOL

Expectedness assessments are made against the approved Reference Safety Information (RSI). The RSI for this trial is specified within each of the candidate specific appendices.

9.7 REPORTING PROCEDURES

All adverse events should be reported. Depending on the nature of the event the reporting procedures below should be followed. Any questions concerning adverse event reporting should be directed to the SCTU in the first instance.

9.7.1 Reporting Details

For all SAEs, SARs and SUSARs either an SAE report on the safety database should be completed with as much detail as possible (including any relevant anonymised treatment forms and/or investigation reports) within 24 hours of site becoming aware of the event. If the safety report cannot be completed on the database, a paper form may be completed, this should be emailed or faxed to the SCTU using the contact details below, within 24 hours of the site becoming aware of the event.

SAE REPORTING CONTACT DETAILS

Please email or fax a copy of the SAE form to
SCTU within 24 hours of becoming aware of the event

Fax: 0844 774 0621 or Email: ctu@soton.ac.uk

FAO: Quality and Regulatory Team

For further assistance: Tel: 023 8120 4138 (Mon to Fri 09:00 – 17:00)

The SAE report asks for nature of event, date of onset, severity, outcome, causality and expectedness. The responsible investigator (or delegate) should assign the seriousness, causality and expectedness of the event with reference to the approved IMP IB/SmPC reference safety information (referenced in the CST protocol) and provide the version used for the assessment.

Additional information should be provided as soon as possible if all information was not included at the time of reporting, but no more than 7 days after initial report.

In addition to the definition above, any suspected transmission via a medicinal product of an infectious agent is also considered an SAE and may be subject to expedited reporting requirements in some countries. Any organism, virus or infectious particle (for example Prion Protein Transmitting Transmissible Spongiform Encephalopathy), pathogenic or non-pathogenic, is considered an infectious agent. Elevations in liver biochemistry that meet Hy's Law criteria are reported as SAEs, using the important medical event serious criterion if no other criteria are applicable.

9.7.2 *Reporting Details International Centres*

All details regarding safety reporting and monitoring for all international sites will be detailed in each CST protocol, along with relevant local and national regulatory guidelines. Follow Up and Post-trial SAEs

The reporting period for each candidate will be outlined in the candidate-specific trial protocol.

All unresolved adverse events should be followed by the investigator until one of the end of trial criteria is met (i.e. lost to follow up, withdrawal etc.). At the last scheduled visit (determined in the candidate-specific trial protocol), the investigator should instruct each participant to report any subsequent event(s) that the participant, or the participant's general practitioner, believes might reasonably be related to participation in this trial. The investigator should notify the trial sponsor of any death or adverse event occurring at any time after a participant has discontinued or terminated trial participation that may reasonably be related to this trial.

9.7.3 *Non-serious AEs*

All adverse events should be recorded in the relevant eCRF and submitted to SCTU.

9.7.4 *Pre-existing Conditions*

Medically significant pre-existing conditions (prior to informed consent) should not be reported as an AE unless the conditions worsens during the trial. The condition, however, must be reported on the Medical History eCRF. Any adverse events that occur after Informed Consent should be recorded on the AE eCRF as per safety reporting section.

9.7.5 *Pregnancy*

If a participant or their partner becomes pregnant whilst taking part in the trial or during a stage where the foetus could have been exposed to an IMP/NIMP, the investigator must ensure that the participant and the participant's healthcare professional are aware that follow up information is required on the outcome of the pregnancy.

Follow-up is of course, dependent on obtaining informed consent for this from the participant (or their partner in the case of male trial participants).

If the participant leaves the area, their new healthcare professional should also be informed.

9.8 SCTU RESPONSIBILITIES FOR SAFETY REPORTING TO REC

SCTU will notify the necessary REC of all SUSARs occurring during the trial according to the following timelines; fatal and life-threatening within 7 days of notification and non-life threatening within 15 days. The responsibility for reporting to non-UK RECs will be delegated to non-UK sites as appropriate.

SCTU will submit the Developmental Safety Update Reports to REC following completion by the CST drug company annually.

9.9 SCTU RESPONSIBILITIES FOR SAFETY REPORTING TO MHRA

See CST protocols for safety reporting responsibilities, however the following guidelines will be strictly adhered to; all SUSARs occurring during the trial according to the following timelines; fatal and life-threatening within 7 days of notification and non-life threatening within 15 days.

SCTU or the organisation described in the CST protocol, will submit the Developmental Safety Update Reports to MHRA following completion by the CST drug company annually.

10 STATISTICS AND DATA ANALYSES

10.1 METHOD OF RANDOMISATION

The default for each candidate trial participants will be block randomisation using a 2:1 allocation to treatment and control. However, randomisation strategy will be assessed for each candidate; this includes the randomisation ratio and use of stratification factors. For any candidate assessed in more than one of the participant groups (A, B and C), stratification by group will be used.

10.2 SAMPLE SIZE

Simulations have shown that around 16 participants are necessary to determine futility or promise of a candidate at a given dose (in efficacy evaluation alone) and between 32 and 40 participants are required across the dose-finding and efficacy evaluation when capping the maximum number of participants contributing to the evaluation of a treatment at 40.

N.B. The CST protocol will give details on the sample size needed for that candidate and a justification for any CST needing n>40.

10.3 STATISTICAL ANALYSIS PLAN (SAP)

All data and appropriate documentation will be stored for a minimum of 25 years after the completion of the trial, including the follow-up period. All analyses will be carried out using STATA, SAS and/or R. Detailed statistical analysis plans will be written and reviewed prior to database freeze for each candidate.

10.3.1 Dose-finding (Phase I)

Study Population

All participants will be accounted for. Definitions of evaluable participants will be determined on a candidate-by-candidate basis.

Analyses

Dose-limiting toxicities will be defined in the CST protocol in advance of recruitment to that CST. Dose-finding data will be summarised descriptively, including baseline characteristics of participants. Dose delivery and toxicities will be reported. Toxicities will be reported by cohort, including type, number, range and worst grade. The parameters of the dose-toxicity model will be described alongside with Bayesian posterior point estimate of the risk of toxicity at each dose and corresponding 95% credible intervals. The models are based on the principles of Mozgunov *et al.*, 2019[9].

Decision Making

A Safety Review Committee will be responsible for confirming candidate-specific trial dose escalations and progress to efficacy evaluation, informed by the dose-toxicity model and safety data. Candidate-specific trial protocol will include full details of this process. A Data Monitoring and Ethics Committee will oversee all the CSTs to ensure patient safety and data integrity, including decision making adherence to CST rules.

10.3.2 *Efficacy evaluation (Phase II)*

All candidate trials will be summarised separately. Each will be reported in accordance with CONSORT guidelines. Each candidate-specific trial is designed to estimate the effect of randomising subjects to an experimental treatment versus randomising participants to a control treatment; in other words, the study's estimand is a "treatment policy" estimand (ICH 2019), with all outcomes up to the end of scheduled follow-up considered relevant, including outcomes after premature withdrawal of study treatment.

Study population

All analyses will be based on an intention-to-treat population as far as possible including participants randomised. Data will be collected up to scheduled end of follow-up, even after a subject withdraws from study treatment, if such withdrawal occurs.

Analyses

Baseline characteristics will be summarized by treatment arm. Continuous data will be presented as means and standard deviations (if data is skewed, medians and ranges will be presented), categorical and binary outcomes will be summarised with frequencies and percentages.

The primary outcome is time to clinical improvement based on the WHO Clinical Progression Scale[8]. This will be analysed using a Cox proportional hazards model under a Bayesian framework, where the distribution for the hazard ratio is updated as data is collected. Covariates to include in the analyses, will be finalised prior to analysis and may change during the course of the study; these are likely to be limited due to anticipated sample size (which will be determined on a per candidate basis, though generally are also not anticipated due to sample size). This will be presented alongside Kaplan-Meier (KM) curves by randomisation group. Patients who die during the study will be censored at day 29 to ensure these patients remain in the number at risk for the duration of the KM curve and count as not achieving the event rather than missing. This is deemed suitable due to the anticipated very low number of people with genuinely missing data for this outcome.

Other time to event outcomes will be analysed in a similar way to the primary outcome. Binary outcomes, such as mortality, will be analysed using logistic regression. No adjustment for multiplicity is currently included to account for potentially multiple treatment comparisons. Secondary endpoints are related to the primary and so are considered supportive analyses.

Consistent with the objective and estimand of the study, a treatment policy strategy will be followed, with the objective of assessing improvement on top of usual care, acknowledging that usual care may change over time. Potential appropriate treatment policy estimands can be worked through with simulations, to assess how changing usual care over time may affect results. Supportive analyses to take account of changes in usual care will include subgroup analyses by type of usual care; and principal stratum analyses; see below for more details of these.

All adverse events and serious adverse events will be summarised by arm with frequencies and percentages.

Subgroup analyses will include a repeat of the primary analysis, and, if numbers allow, may include the below; it is likely that these will only be considered in AGILE if a candidate is assessed in phase II.

- By type of usual care and if applicable by time of introduction of new usual care;
- Of the principal stratum of subjects who would always receive new usual care irrespective of randomized treatment and, separately, of the principal stratum of subjects who would never receive the new usual care irrespective of randomized treatment (Rubin 1998; SAS macro available and to be made downloadable at missingdata.org.uk);
- By underlying health condition (cardiovascular; diabetes; any of chronic respiratory disease, hypertension or cancer; obesity; none of the above (Novel coronavirus pneumonia emergency response epidemiology team (2020));

- By age group (up to 69 years of age; more than 69 years of age) (Novel coronavirus pneumonia emergency response epidemiology team (2020)).

11 REGULATORY

11.1 CLINICAL TRIAL AUTHORISATION

This trial has a Clinical Trial Authorisation from the UK Competent Authority the Medicines and Healthcare products Regulatory Agency (MHRA).

12 ETHICAL CONSIDERATIONS

The trial will be conducted in accordance with the recommendations for physicians involved in research on human subjects adopted by the 18th World Medical Assembly, Helsinki 1964 as revised and recognised by governing laws and EU Directives. Each participant's consent to participate in the trial should be obtained after a full explanation has been given of treatment options, including the conventional and generally accepted methods of treatment. The right of the participant to refuse to participate in the trial without giving reasons must be respected.

After the participant has entered the trial, the clinician may give alternative treatment to that specified in the candidate-specific trial protocol, at any stage, if they feel it to be in the best interest of the participant. However, reasons for doing so should be recorded and the participant will remain within the trial for the purpose of follow-up and data analysis according to the treatment option to which they have been allocated. Similarly, the participant remains free to withdraw at any time from protocol treatment and trial follow-up without giving reasons and without prejudicing their further treatment.

The trial protocol and each candidate-specific trial protocol has received the favourable opinion of a Research Ethics Committee.

12.1 CONFIDENTIALITY

SCTU will preserve the confidentiality of participants taking part in the trial. The investigator must ensure that participant's anonymity will be maintained and that their identities are protected from unauthorised parties. On eCRFs participants will not be identified by their names, but by an identification code.

13 SPONSOR

SCTU, Chief Investigator and other appropriate organisations have been delegated specific duties by the Sponsor and this is documented in the trial task allocation matrix.

The duties assigned to the trial sites (NHS Trusts or others taking part in this trial) are detailed in the Non-Commercial Agreement.

13.1 INDEMNITY

University of Liverpool holds insurance against claims from participants for harm caused by their participation in this clinical study. However, the treating hospital continues to have a duty of care to the participant and the Sponsor does not accept liability for any breach in the hospital's duty of care, or any negligence of the part of hospital employees. In these cases, clinical negligence indemnification will rest with the participating NHS Trust or Trusts under standard NHS arrangements.

The University of Southampton's public and professional indemnity insurance policy provides an indemnity to UoS employees for their potential liability for harm to participants during the conduct of the research. This does not in any way affect an NHS Trust's responsibility for any clinical negligence on the part of its staff.

For trials outside the UK, indemnity arrangements will be defined within clinical trial agreements between the University of Liverpool (as Sponsor) and the trial site. These will be amongst the essential criteria for Sponsor 'Green Light' to open the study.

13.2 FUNDING

Each CST will secure funding with additional finacial support from the NIHR Southampton Clinical Trials Unit.

The individual CST protocols will detail if participants will be paid for participation in this trial.

14 TRIAL OVERSIGHT GROUPS

The day-to-day management of the trial will be co-ordinated through the SCTU and oversight will be maintained by the Trial Management Group, the Trial Steering Committee the Data Monitoring and Ethics Committee and Candidate-Specific Safety Review Committees (SRC).

14.1 TRIAL MANAGEMENT GROUP (TMG)

An AGILE TMG will responsible for overseeing progress of the trials within the trial platform, including both the clinical and practical aspects. The co-chairs of the TMG will be the Chief Investigator of the trial and Director of SCTU.

The AGILE TMG charter defines the membership, terms of reference, roles, responsibilities, authority, decision-making and relationships of the TMG, including the timing of meetings, frequency and format of meetings and relationships with other trial committees. This will include the clinical leads for each CST protocol.

14.2 TRIAL STEERING COMMITTEE (TSC)

The TSC act as the oversight body of the AGILE trial platform on behalf of the Sponsor and Funder. The TSC will meet when required for each CST but at least twice a year. The majority of members of the TSC, including the Chair, should be independent of the trial.

The AGILE TSC charter defines the membership, terms of reference, roles, responsibilities, authority, decision-making and relationships of the TSC, including the timing of meetings, frequency and format of meetings and relationships with other trial committees.

14.3 DATA MONITORING AND ETHICS COMMITTEE (DMEC)

The AGILE trial will have an overarching DMEC for all CSTs. The aim of the DMEC is to safeguard the interests of trial participants, monitor the main outcome measures including safety and efficacy, and monitor the overall conduct of the trial.

The AGILE DMEC charter defines the membership, terms of reference, roles, responsibilities, authority, decision-making and relationships of the DMEC, including the timing of meetings, methods of providing information to and from the DMEC, frequency and format of meetings, statistical issues and relationships with other trial committees.

14.4 CANDIDATE-SPECIFIC TRIAL SAFETY REVIEW COMMITTEE (SRC)

Each CST will have an SRC (if required). The SRC will meet to review safety data at agreed time points agreed during phase I, usually when each cohort of patients have been treated with the candidate cohort target dose at least once. If required, the SRC may meet before these time points. Each CST protocol will outline full details of the CST SCR.

15 DATA MANAGEMENT

Participant data will be entered remotely at site to Medidata Rave EDC via tablet or other suitable access to the SCTU URL and retained in accordance with the current Data Protection Regulations. The PI is responsible for ensuring the accuracy, completeness, and timeliness of the data entered.

All participant data collected is pseudo anonymised, by assigning each participant a participant identifier code which is used to identify the participant during the trial and for any participant- specific clarification between SCTU and site. The site retains a participant identification code list which is only available to site staff.

The Master Participant Information Sheet and Informed Consent Form will outline the participant data to be collected and how it will be managed or might be shared; including handling of all Patient Identifiable Data (PID) and sensitive PID adhering to relevant data protection law.

Trained personnel with specific roles assigned will be granted access to the electronic case report forms (eCRF). eCRF completion guidelines will be provided to the investigator sites to aid data entry of participant information.

Only the Investigator and personnel authorised by them should enter or change data in the eCRFs. When requested, laboratory data must be transcribed, with request investigator observations entered into the eCRF. The original laboratory reports must be retained by the Investigator for future reference.

A Data Management Plan (DMP) providing full details of the trial specific data management strategy for the trial will be available and a Trial Schedule with planned and actual milestones, CRF tracking and central monitoring for active trial management created.

Data queries will either be automatically generated within the eCRF, or manually raised by the trial team, if required. All alterations made to the eCRF will be visible via an audit trail which provides the identity of the person who made the change, plus the date and time.

At the end of the trial after all queries have been resolved and the database frozen, the PI will confirm the data integrity by electronically signing all the eCRFs. The eCRFs will be archived according to SCTU policy and a PDF copy including all clinical and Meta data returned to the PI for each participant.

Data may be requested from the Data Access Committee at SCTU. Any request will be considered on a monthly basis. Data may also be downloaded at regular, pre-defined timepoints to visualisation and analytics tools to aid evaluation and decision making, looking for trends and underlying signals in the data.

16 DATA SHARING REQUESTS FOR RESULTS THAT ARE AVAILABLE IN THE PUBLIC DOMAIN

In order to meet our ethical obligation to responsibly share data generated by interventional clinical trials, SCTU operate a transparent data sharing request process. As a minimum, anonymous data will be available for request from three months after publication of an article, to researchers who provide a completed Data Sharing request form that describes a methodologically sound proposal, for the purpose of the approved proposal and if appropriate a signed Data Sharing Agreement. Data will be shared once all parties have signed relevant data sharing documentation.

Researchers interested in our data are asked to complete the Request for Data Sharing form (CTU/FORM/5219) [template located on the SCTU web site, www.southampton.ac.uk/ctu] to provide a brief research proposal on how they wish to use the data. It will include; the objectives, what data are requested, timelines for use, intellectual property and publication rights, data release definition in the contract and participant informed consent etc. If considered necessary, a Data Sharing Agreement from Sponsor may be required.

17 MONITORING

17.1 CENTRAL MONITORING

Data stored at SCTU will be checked for missing or unusual values (range checks) and checked for consistency within participants over time. Any suspect data will be returned to the site in the form of data queries. Data query forms will be produced at SCTU from the trial database and sent either electronically or through the post to a named individual (as listed on the site delegation log). Sites will respond to the queries providing an explanation/resolution to the discrepancies and return the data query forms to SCTU within the required timeframe. The forms will then be filed along with the appropriate CRFs and the appropriate corrections made on the database.

Refer to each candidate-specific trial (CST) protocol for informed consent procedure, which may include paper or e-consent.
SCTU will conduct central monitoring of consent forms. Paper informed consent forms will be scanned and securely transferred to SCTU by means of SafeSend https://safesend.soton.ac.uk/, encrypted email, or secure nhs.net email.

There are a number of monitoring principles in place at SCTU to ensure reliability and validity of the trial data, which are detailed in the trial monitoring plan.

17.2 CLINICAL SITE MONITORING

Due to COVID-19 being a highly contagious pandemic it is expected that no or very limited site monitoring will be conducted - including Source Data Verification (SDV). The AGILE trial will use an enhanced central monitoring process as detailed in the AGILE risk assessment and monitoring plan. The monitoring plan for the trial may be modified for each trial compound as required.

17.3 SOURCE DATA

Source documents are where data are first recorded, and from which participants' CRF data are obtained. These include, but are not limited to, hospital records (from which medical history and previous and concurrent medication may be summarised), clinical and office charts, laboratory and pharmacy records, diaries, microfiches, radiographs, and correspondence.

17.4 AUDITS AND INSPECTIONS

The trial may be participant to inspection and audit by the sponsor (under their remit as Sponsor), SCTU (as the Sponsor's delegate) and other regulatory bodies to ensure adherence to the principles of GCP, Research Governance Framework for Health and Social Care, applicable contracts/agreements and national regulations. These could also include companies who are providing candidate drug which will be specified in the candidate specific appendices.

18 RECORD RETENTION AND ARCHIVING

Trial documents will be retained in a secure location during and after the trial has finished.

The PI or delegate must maintain adequate and accurate records to enable the conduct of the trial to be fully documented and the trial data to be subsequently verified. After trial closure the PI will maintain all source documents and trial related documents. All source documents will be retained for a period of 25 years following the end of the trial. If the trial compound is deemed to be an Advanced Therapy Investigational Medicinal Product (ATIMP), then all relevant documentation will be retained for 30 years

Sites are responsible for archiving the ISF and participants' medical records.

The Sponsor is responsible for archiving the TMF and other relevant trial documentation.

19 PUBLICATION POLICY

Data for each candidate will be analysed and published as soon as possible to ensure rapid availability of results to avoid duplication from others across the world. To ensure this, in the first instance these results may be published in a non-standard format presenting headline results as soon as possible on SCTU website (for example) followed by a formal publication.

Individual investigators may not publish data concerning their participants that are directly relevant to questions posed by the trial until the Trial Management Group (TMG) has published its report. The TMG will form the basis of the Writing Committee and advise on the nature of publications. All publications shall include a list of investigators, and if there are named authors, these should include the Chief Investigator, Co-Investigators, Trial Manager, and Statistician(s) involved in the trial. Named authors will be agreed by the CI and Director of SCTU. If there are no named authors then a ‘writing committee’ will be identified.

20 REFERENCES

1. Zhu N, Zhang D, Wang W, et al. A Novel Coronavirus from Patients with Pneumonia in China, 2019. N Engl J Med 2020.
2. Huang C, Wang Y, Li X, et al. Clinical features of patients infected with 2019 novel coronavirus in Wuhan, China. Lancet 2020.
3. European Medicines Agency: Treatments and vaccines for Covid 19: authorised medicines; Link: Treatments and vaccines for COVID-19: authorised medicines | European Medicines Agency (europa.eu) visited on 10.02.2021.
4. Chen N, Zhou M, Dong X, et al. Epidemiological and clinical characteristics of 99 cases of 2019 novel coronavirus pneumonia in Wuhan, China: a descriptive study. Lancet 2020.
5. Wang D, Hu B, Hu C, et al. Clinical Characteristics of 138 Hospitalized Patients With 2019 Novel Coronavirus-Infected Pneumonia in Wuhan, China. JAMA 2020.
6. Cao et al. (2020). A Trial of Lopinavir–Ritonavir in Adults Hospitalized with Severe Covid-19. *New England Journal of Medicine*. DOI: 10.1056/NEJMoa2001282
7. Simon et al. (1985). Randomized phase II clinical trials. *Cancer Treatment Reports*. Vol 69(12), pp 1375-81
8. WHO Working Group on the Clinical Characterisation and Management of COVID-19 infection (2020). A minimal common outcome measure set for COVID-19 clinical research. *Lancet Infect Dis* 2020;20: e192–97.
9. Mozgunov, P., Jaki, T. and Paoletti, X. (2019) Randomized dose-escalation designs for drug combination cancer trials with immunotherapy. Journal of Biopharmaceutical Statistics, 29, 359–377.

21 MASTER PROTOCOL ACCESS

The Master protocol can be accessed via the AGILE trial website at www.agiletrial.net. Each candidate specific (CST) Protocol will be provided to sites separately.

22 SUMMARY OF SIGNIFICANT CHANGES TO THE PROTOCOL

Protocol date and version	Summary of significant changes
v1.0 14-Apr-2020	n/a v1.0
v2.0 16-Apr-2020	• Section 5.1 Updates to legal representative informed consent
v3.0 24-Apr-2020	• Section 3 qSOFA added as alternative endpoint for ventilated patients (assessment already in protocol)<br>• Section 3 Mortality endpoint clarified measured from randomisation (not registration)<br>• Section 4.2.2 Trial schema for dose finding and efficacy evaluation corrected to state assessment for efficacy would be done in day 29 not 21<br>• Section 8.1 NEWS and qSOFA assessments removed (screening) (included in error)<br>• Section 8.2 Clarification added regarding follow up assessments if patients are discharged

	• Section 8.2 Addition that if WHO assessment done over the phone at Day 15, 29 and 36 date patient last changed one point on the scale to be collected (to allow for primary endpoint (time to event) data to be collected)<br>• 8.2.6 Corrected ± window for Day 29 (2 days not 5)<br>• Section 9.2 AE/SAE reporting windows updated to refer to CST protocols<br>• Section 9.3 Clarification that for onwards reporting SCTU will code CTCAE terms entered by site to MedDRA<br>• Section 9.9 Clarification that drug companies will write DSURs
v4.0 01-May-2020	• Change of Chief Investigator from Prof Tom Wilkinson to Prof Saye Khoo
v5.0 07-May-2020	• Section 5.1: Updates to the legal representative informed consent process and removed the need for a witness ICF
v6.0 15-Jun-2020	• Change of trial name to AGILE: Seamless Phase I/IIa Platform for the Rapid Evaluation of Candidates for COVID-19 treatment<br>• Change of Sponsor from University Hospital Southampton NHS Foundation Trust to University of Liverpool (including updates to indemnity)<br>• Update of safety reporting to refer to CST protocols
v7.0 08-Sep-2020	• WHO clinical severity score 9-point ordinal scale assessment replace with WHO Clinical Progression Scale.<br>• Group A (severe disease) definition amended to reflect updated WHO Clinical Progression Scale.<br>• References amended to other national platforms to reflect change in landscape.<br>• References to emerging clinical trial data included.
V8<br>20-Jan-2021	• ISRCTN reference added<br>• Section 1.1: Trial Synopsis - Addition of criteria for site selection<br>• Section 1.2: Trial Schema – Updated<br>• Section 2.1.3: Addition of circumstances when it would be appropriate to bypass Phase Ib and go straight to Phase IIa<br>• Addition of Group C healthy volunteers undertaking Phase Ia trials (relevant sections updated throughout protocol)<br>• Addition of reference to international trial sites (relevant sections updated throughout protocol)<br>• Section 5.1: updated to include recognition of local guidelines and established practice for recruitment and consent of unconscious patients outside of the UK<br>• Section 5.3 – Update to exclusion criteria 7<br>• Section 10.1 – Update to method of randomisation<br>• Section 10.3.1 – Reference added<br>• Section 14.2 – Update to TSC meeting requirements<br>• Section 17.1 – Updated to include paper or e-consent options. Addition of process for secure transfer of consent forms to SCTU for central monitoring<br>• Section 20 – Reference added
V9<br>17-Feb-2021	• ClinicalTrials.Gov reference added<br>• Trial management email agile@soton.ac.uk added, removal of agile2@soton.ac.uk reference<br>• Addition of Sponsor signature on Protocol front page<br>• List of abbreviations: addition of UK-CTAP and removal of SAB<br>• Section 1.1: Trial synopsis – further clarification of healthy volunteers group

	• Section 1.1: Trial synopsis – open-label wording removed to align with CST5 double-blinded design and leave broad for future CSTs.<br>• Section 1.1: Trial synopsis – reference to SAB removed and updated with UK-CTAP<br>• Section 1.1: Trial synopsis – Co-primary endpoints updated for healthy volunteers Group C<br>• Section 2.1: Update to authorised medicines for Covid 19<br>• Section 2.1.1: Removal of SAB and insertion of UK-CTAP<br>• Section 4.1: Study Design – open-label wording removed to align with CST5 double-blinded design and leave broad for future CSTs<br>• Section 20: Reference added<br>• Section 21: Removal of CST protocol link on website and update to just master protocol access
